# *TaPYL4*, an ABA receptor gene of wheat, positively regulates plant drought adaptation through modulating the osmotic stress-associated processes

**DOI:** 10.1186/s12870-022-03799-z

**Published:** 2022-09-01

**Authors:** Yanyang Zhang, Yingjia Zhao, Tianjiao Li, Chenyang Ni, Le Han, Pingping Du, Kai Xiao

**Affiliations:** 1 State Key Laboratory of North China Crop Improvement and Regulation, Baoding, Hebei 071001 People’s Republic of China; 2 College of Agronomy, Hebei Agricultural University, Baoding, 071001 People’s Republic of China; 3 Key Laboratory of Crop Growth Regulation of Hebei Province, Baoding, People’s Republic of China

**Keywords:** Wheat (*Triticum aestivum* L.), abscisic acid receptor, gene expression, drought stress, stress-defensive physiological indices

## Abstract

**Background:**

Abscisic acid receptors (ABR) involve transduction of the ABA signaling in plants, impacting largely on stress-defensive physiological processes and plant osmotic stress response. In this study, we characterized *TaPYL4*, a gene of ABR family in *T. aestivum*, in mediating plant drought tolerance given scarcity of functional characterization on wheat ABR members thus far.

**Results:**

TaPYL4 harbors nine conserved domains shared by its PYL counterparts, targeting onto plasma membrane and nucleus after endoplasmic reticulum assortment. TaPYL4 interacts with TaPP2C2 whereas the latter with TaSnRK2.1, which establish a core module of the ABA signaling pathway. *TaPYL4* expression was upregulated in root and aerial tissues upon drought stress. Overexpressing *TaPYL4* conferred plants improved growth traits whereas knockdown expression of target gene alleviated growth feature compared with wild type under drought treatment. The *TaPYL4*-enhanced drought adaptation associates gene function in positively regulating stomata movement, osmolyte biosynthesis, and root system architecture (RSA) establishment. Expression analysis on the P5CS family genes involving proline biosynthesis indicated that *TaP5CS1* exerts critical roles in promoting osmolytes accumulation in drought-challenged *TaPYL4* lines. *TaPIN9*, a PIN-FORMED gene modulating cellular auxin translocation, was validated to function as a crucial mediator in defining RSA establishment underlying *TaPYL4* regulation. Transcriptome analysis revealed that *TaPYL4* controls transcription of numerous genes, which impact on physiological processes associated with ‘*biological process*’, ‘*molecular component*’, and ‘*cellular process*’. Moreover, the differentially expressed genes mediated by *TaPYL4* were closely related to stress defensive pathways.

**Conclusions:**

Our investigation suggested that *TaPYL4* acts as a positive regulator in plant drought tolerance and a valuable target for engineering drought-tolerant cultivars in *T. aestivum*.

**Supplementary Information:**

The online version contains supplementary material available at 10.1186/s12870-022-03799-z.

## Background

Drought stress negatively regulates the plant growth and development by inhibiting various biochemical and physiological processes [1]. On the other hand, plants have evolved a suite of effective mechanisms to acclimate them to adverse effects initiated by drought [2]. Upon perception of drought signaling, the signal transduction systems in root tissue transduce the stress cues across whole plant through distinct signaling transduction pathways [3, 4]. Thus far, a large set of signaling members functional in the plant drought signal transduction system have been documented [5–7], which contribute to the drought adaptation of plants by regulating transcription efficiency of genes in families of transcription factor (TF) [8], ion channel and transporter [9], and antioxidant enzyme [10], etc., mainly through the abscisic acid (ABA)-dependent pathways [11].

The ABA molecule modulates diverse physiological processes associated with plant growth, development and stress responses. Upon osmotic stresses, the ABA contents in root tissue are swiftly elevated due to the altered transcription of genes regulating the ABA biosynthesis [12]. The ABA molecules induced in the tissue were further transported and distributed throughout various organs mediated by corresponding cellular import transporters, finally eliciting the plant response and adaptation to distinct osmotic stressors [12].

Perception of the ABA signaling upon abiotic stresses is mediated mainly by the ABA-dependent pathway. Of which, abscisic acid (ABA) receptors (ABRs) are functional as the critical core components. In plant species, ABRs are present as a family of proteins, possessing the ability to interact with their partners referred to as phosphatases in clade A PP2C. Thus far, the members of ABR family, such as pyrabactin resistance (PYR) 1, PYR1-like, and proteins in the PYL family, have been characterized in the model plant *A. thaliana* [13–15]. Protein structural analysis on them has defined the ternary crystal features among ABRs, the substrate ABA, and the PP2C proteins [16, 17]. These findings provide valuable information in elucidating the mechanisms as to the ABA signaling perception for osmotic stresses in plant species.

To date, the molecular processes underlying the ABA-mediated plant drought response have been explored in depth given powerful research approach and valuable mutant materials. Under osmotic stress conditions (i.e., drought), the ABRs (PYR/PYL proteins) bound to ABA molecules induced and then interact with PP2Cs, removing the inhibition of PP2C activities on the kinase proteins of SnRK2 family. The activation of the SnRK2 family members phosphorylates/activates the transcription factors (TFs), membrane channels, and transcription of the stress-defensive genes essential in plant drought tolerance [9]. Therefore, further characterization on the module ABR/PP2C/SnRK2 of the ABA signaling pathway can deepen understanding the mechanisms of plant osmotic stress response underlying the ABA-dependent pathways.

Currently, the ABR family members have been extensively investigated in various plant species, especially in model plant *A. thaliana* [18]. Additionally, the components constituting the core functional module of ABA signaling pathway, such as the partners interacting with ABRs (i.e., PP2C proteins) and those with PP2C proteins (SnRK2 members) have also been characterized in Arabidopis [19]. Moreover, functional characterization on the members in ABA receptor family have indicated that distinct genes of this family act as critical mediators in plant drought adaptation, associating with their roles in regulating the drought stress-responsive processes. For example, the transgenic plants overexpressing *RCAR11-RCAR14*, four genes in the ABA receptor subfamily III in Arabidopsis, were improved on plant drought resistance by increasing transcription of a suite of stress-responsive genes that contributes to improved pant water-use efficiency under drought stress conditions [10]. Overexpression of rice ABA receptor gene *PYL10* resulted in higher ABA levels in plants due to upregulated expression of genes for ABA biosynthesis including *ZEP1*, *NCED1*, *NCED2*, *NCED3*, and *NCED4*, enhancing yield formation of the drought-challenged plants through maintaining higher RWC, membrane stability index, chlorophyll content, and accumulated lower amount of MDA and H_2_O_2_ compared with WT plants [20]. The transgenic lines overexpressing *OsPYL6*, another ABA receptor gene in *O. sativa*, display enhanced ABA hypersensitivity during germination, promoted total root length of seedlings and enhanced ABA accumulation in plants, positively regulating the expression of stress-responsive genes and plant dehydration tolerance by significantly reducing plant transpiration [21]. These findings corroborate that distinct members of the ABA receptor family act as essential determinants in enhancing plant drought tolerance through modulating stress-defensive biological process in the ABA-dependent pathways.

In *T. aestivum*, a set of genes encoding proteins of the PYR/PYL families have been documented [22]. However, detailed characterizations on the components of the core ABA signaling module are still to be determined. Additionally, the mechanisms underlying the ABA receptor-mediated plant drought tolerance are also largely elusive in wheat. In this study, we characterized *TaPYL4* (*TaPYL4-4A*), a member of the ABR family in *T. aestivum* shown to be upregulated in transcription under drought condition in our previous expression analysis (unpublished data), in mediating the plant response to drought stress. Our findings suggest that *TaPYL4* acts as one of critical mediators in plant drought adaptation by improving osmotic stress-associated physiological processes. This ABR gene also regulates the transcription of genes at global level functional in modulating various physiological processes mainly through the stress defensive pathway.

## Results

### Characterization of *TaPYL4*

**Fig. 1:**
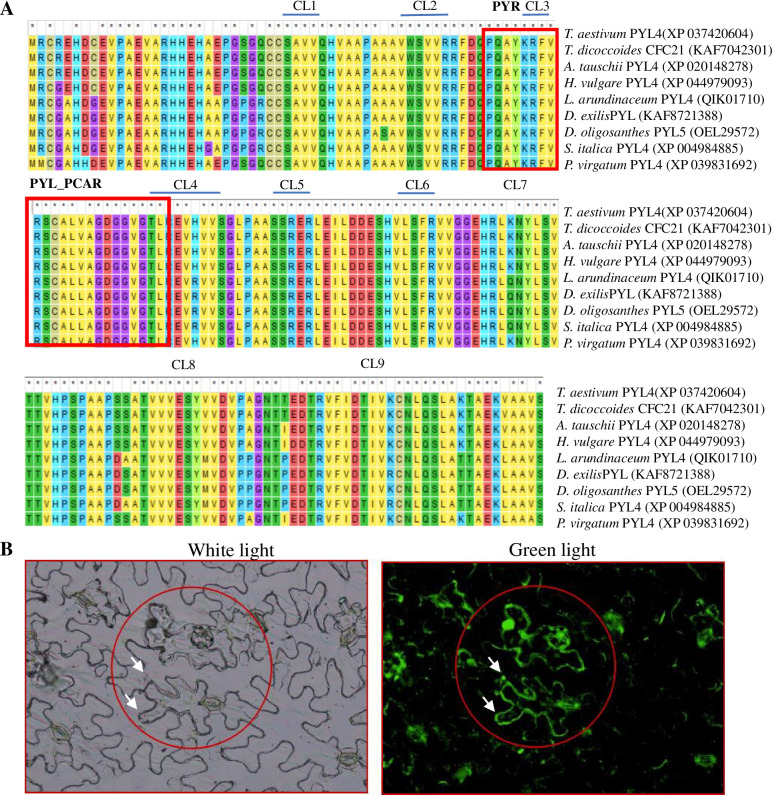
Characterization of the TaPYL4 protein. **A** ClustalW results among the TaPYL4 protein and its counterparts in plant species; **B** the GFP signals in transgenic epidermal cells harboring fusion *TaPYL4-GFP* detected under fluorescent microscope. In **A** CL1 to CL 9, nine conserved motifs specified by PYL proteins; PYR and PYL_PCAR, the conserved domains harbored in ABA receptors. In **B** two typical epidermal cells are circled and arrows point to plasma membrane and nucleus, respectively

*TaPYL4* has a full length cDNA of 1112 bp, encoding a 179-aa polypeptide with a molecular mass of 14.93 kDa and an isoelectric point (pI) of 5.10. The TaPYL4 protein harbors nine conserved domains same as its plant counterparts (i.e., CL1 to CL9), which are involved in the binding to ABA molecule and in the interaction with its the downstream partners, such as PP2C proteins (Fig. 1A). At nucleic acid level, *TaPYL4* shares high similarities to its homologous genes distributed in various plant species, including *F. arundinacea PYL4* (with cDNA sequence identity of 93.89%, MN259578), *S. viridis PYL4* (90.88%, MG766908), *Z. mays PYL4* (90.84%, NM 001319727), *Z. mays PYL7* (90.84%, KJ855102), *Z. japonica PYL3* (91.42%, KY475605), and *F. elataPYL3* (92.63%, KY475599) (Additional file 1), suggesting its nature to be one of the PYL family members in *T. aestivum*. Based on an experiment to define the sub-cellular position of target-GFP fusion at cellular level, the signals derived from TaPYL4-GFP in epidermal cells of *N. benthamiana* were confined onto plasma membrane and nucleus (Fig. 1B, Additional file 2). These results suggested that TaPYL4 targets onto both above locations after ER assortment where it exerts distinct biological roles.

### The components constitutes ABA signaling module with TaPYL4

**Fig. 2:**
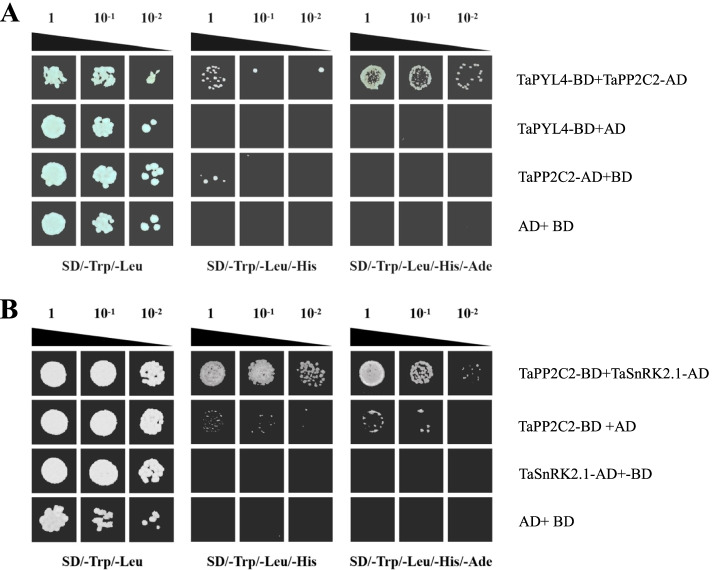
Yeast two-hybridization assays for detection of components in core ABA signaling module among TaPYL4 and its downstream partners. **A** protein interaction between TaPYL4 and TaPP2C2; **B** protein interaction between TaPP2C2 and TaSnRK2.1. TaPP2C2, a member of the clade A PP2C family in *T. aestivum*. TaSnRK2.1, a member of the SnRK2 family in *T. aestivum*

Based on yeast two-hybridization assays, the component in clade A class PP2C family interacting with TaPYL4 was identified. Results indicated that TaPYL4 specifically interacted with the PP2C member TaPP2C2 (Fig. 2A). Similar assays were performed to determine the partner to be involved in interacting with TaPP2C2. It was shown that TaSnRK2.1, a member of the SnRK2 family in *T. aestivum*, specifically interacted with this wheat PP2C protein mentioned above (Fig. 2B). Therefore, the results in our protein-protein interaction analysis indicated that TaPYL4 constitutes a core ABA signaling module with components of TaPP2C2 and TaSnRK2.1, namely, TaPYL4-TaPP2C2-TaSnRK2.1. This module was speculated to play crucial roles in the ABA signal pathways through transduction of signaling initiated by distinct internal or environmental cues through the protein phosphorylation mechanism.

### Expression patterns of *TaPYL4* under drought stress conditions

**Fig. 3:**
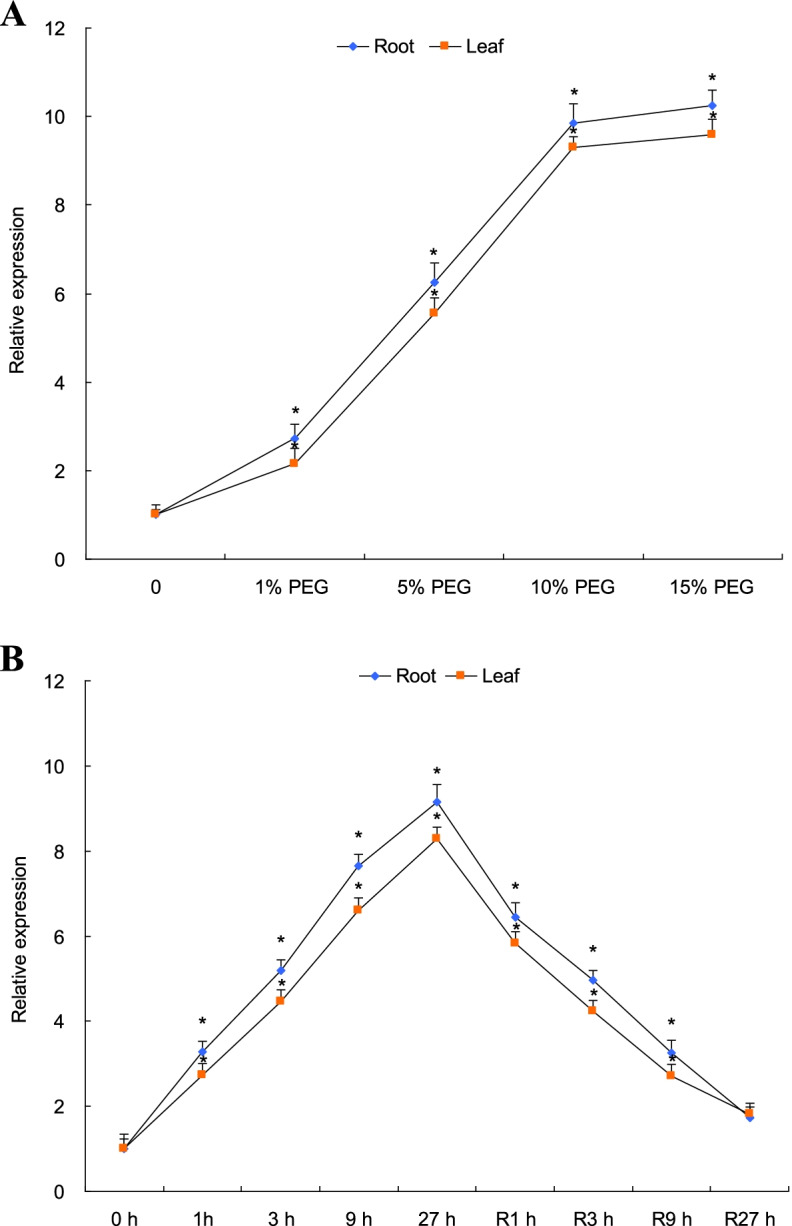
Expression patterns of *TaPYL4* in roots and aerial tissues under drought stress condition. **A** transcripts of *TaPYL4 *in tissues upon modified external PEG concentrations; **B** transcripts of *TaPYL4* under drought as well as normal recovery treatments. In **B** 0 h, time point prior to drought treatment (control); 1 h, 3 h, 9 h, and 27 h, times following drought condition; R1 h, R3 h, R9 h, and R 27 h, times following recovery growth condition. Values shown are averages derived from triplicate results and error bars indicate standard errors with symbol * to represent statistically significant compared with control (without PEG in **A** and 0 h in **B**) (*P* < 0.05). Expression levels shown are ratios of the transcripts between target gene and the reference *Tatubulin*

The transcripts of *TaPYL4* in tissues of root and aerial tissues were evaluated to characterize the expression patterns of target gene in response to drought stress. Upon drought stress treatment, the expression levels of *TaPYL4* in both tissues were significantly upregulated following the intensified extent of drought stressor, showing to be gradually elevated along with the increased polyethylene glycol (PEG) in growth media (0 to 15% (w/v) PEG-6000) (Fig. 3A). Additionally, under drought stress condition (10% PEG-6000), the *TaPYL4* expression in tissues displayed a temporal-dependent pattern in response to drought stressor within a 27 h regime, reaching peak level at end of the treatment. Moreover, the induced transcripts of target gene in tissues under drought stress condition were gradually recovered following a 27 h of normal recovery condition (Fig. 3B). These results together suggested that *TaPYL4* sensitively responds to drought stress at transcriptional level, mediating plant drought response through its spatiotemporal mode of transcription in response to drought stressors.

### Growth properties of *TaPYL4* transgenic lines under drought treatment

**Fig. 4:**
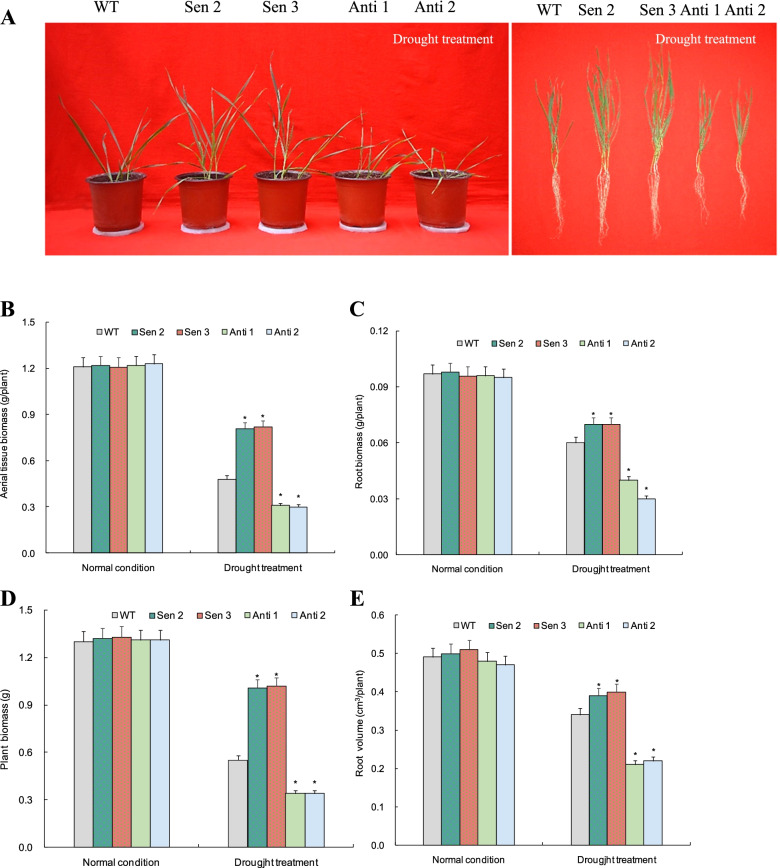
Phenotypes and growth traits in *TaPYL4* transgenic lines under drought treatment. **A** phenotypes under normal condition and drought stress conditions; **B** aerial tissue biomass; **C** root biomass; **D** plant biomass; **E** root volume. WT, wild type; Sen 2 and Sen 3, two lines with *TaPYL4* overexpression; Anti 1 and Anti 2, two lines with knockdown expression of *TaPYL4*. In **B** to **E** data shown are average plus standard error with symbol * to represent statistically significant compared with WT (*P* < 0.05)

Sen 2 and Sen 3, two transgenic lines at T3 generation with more *TaPYL4* transcripts and Anti 1 and Anti 2, two lines with significant repression of target expression with one copy inserted (Additional file 3), were selected to define the gene function in mediating plant drought tolerance. Under normal growth condition, all of transgenic lines (i.e., Sen 2, Sen 3, Anti 1, and Anti 2) were comparable on phenotypes, biomass of aerial tissue and root, and root volumes with the WT plants (Fig. 4A-E). Under drought stress treatment, however, the transgenic lines were shown to be dramatically modified on growth traits mentioned with respect to WT. Of which, Sen 2 and Sen 3 were much more improved on phenotypes (Fig. 4A), biomass of aerial tissue (Fig. 4B), root biomass (Fig. 4C), plant biomass (Fig. 4D), and root volumes (Fig. 4E) than WT plants. In contrast, compared with WT, Anti 1 and Anti 2 drastically alleviated phenotypes (Fig. 4A), biomass of aerial tissue (Fig. 4B), root biomass (Fig. 4C), plant biomass (Fig. 4D), and root volumes (Fig. 4E) in plants treated by drought stress. The significantly modulated growth behavior was shown in the drought-challenged transgenic lines, suggesting that *TaPYL4* plays an important role in mediating plant adaptation to drought stress.

### Stress response-associated physiological traits of *TaPYL4* transgenic lines

**Fig. 5:**
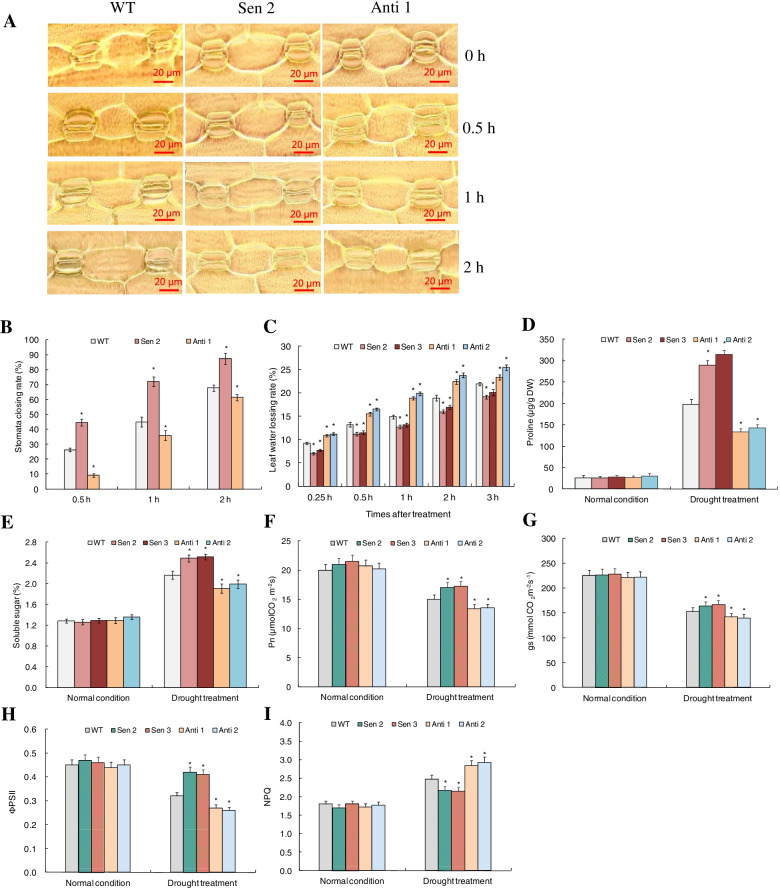
Stomata characterization and osmolyte accumulation as well as photosynthetic parameters of *TaPYL4* transgenic lines upon drought stress. **A** stomata behaviors; **B** stomata closing rates; **C** leaf water closing rates; **D** proline contents; **E** soluble sugar contents; **F** Pn; **G** gs; **H** ΦPSII; **I** NPQ. WT, wild type; Sen 2 and Sen 3, two lines overexpressing *TaPYL4*; Anti 1 and Anti 2, two lines with knockdown expression of *TaPYL4*. In **B** data shown are those relative to 0 h. In **B** to **I** data shown are averages and symbol * represents statistically significant compared with WT (*P* < 0.05)

A suite of stress-associated traits and photosynthetic parameters were measured in transgenic lines (Sen 2, Sen 3, Anti 1, and Anti 2) under drought stress conditions to understand the physiological processes mediated by *TaPYL4*. In line with the growth traits described above, all the transgenic lines were comparable on the stress-associated traits (i.e., stomata closing rate (SCR), leaf water losing rate (WSR), and contents of proline and soluble sugar) (Fig. 5A-E), and the photosynthetic parameters (i.e., photosynthetic rate (Pn), stomatal conductance (gs), ΦPSII, and NPQ) (Fig. 5F-I) with WT plants under normal growth condition. Under drought stress treatment, compared with WT, Sen 2 and Sen 3 displayed promoted SCR (Fig. 5A-B, Additional file 4), slowed WLR (Fig. 5C), increased contents of osmolytes (proline and soluble sugar) (Fig. 5D-E), and enhanced Pn (Fig. 5F), (Fig. 5G), and ΦPSII (Fig. 5H), and reduced NPQ (Fig. 5I). In contrast, Anti 1 and Anti 2 drastically alleviated the stress-associated traits and the photosynthetic parameters, displaying lower SCR (Fig. 5A-B, Additional file 4), higher elevated leaf WLR (Fig. 5C), less contents of osmolytes (Fig. 5D-E), and more alleviated photosynthetic function (lower Pn, gs, and ΦPSII, and higher NPQ values) than the WT plants (Figs. 5F-I). The physiological traits associated with plant stress response were in concordance with growth features of the transgenic lines under drought treatment, suggesting that the *TaPYL4*-mediated drought tolerance of plants ascribes partly to the gene function in improving the stress responsive-associated physiological processes.

### Expression patterns of P5CS and PIN-FORMED family genes

**Fig. 6:**
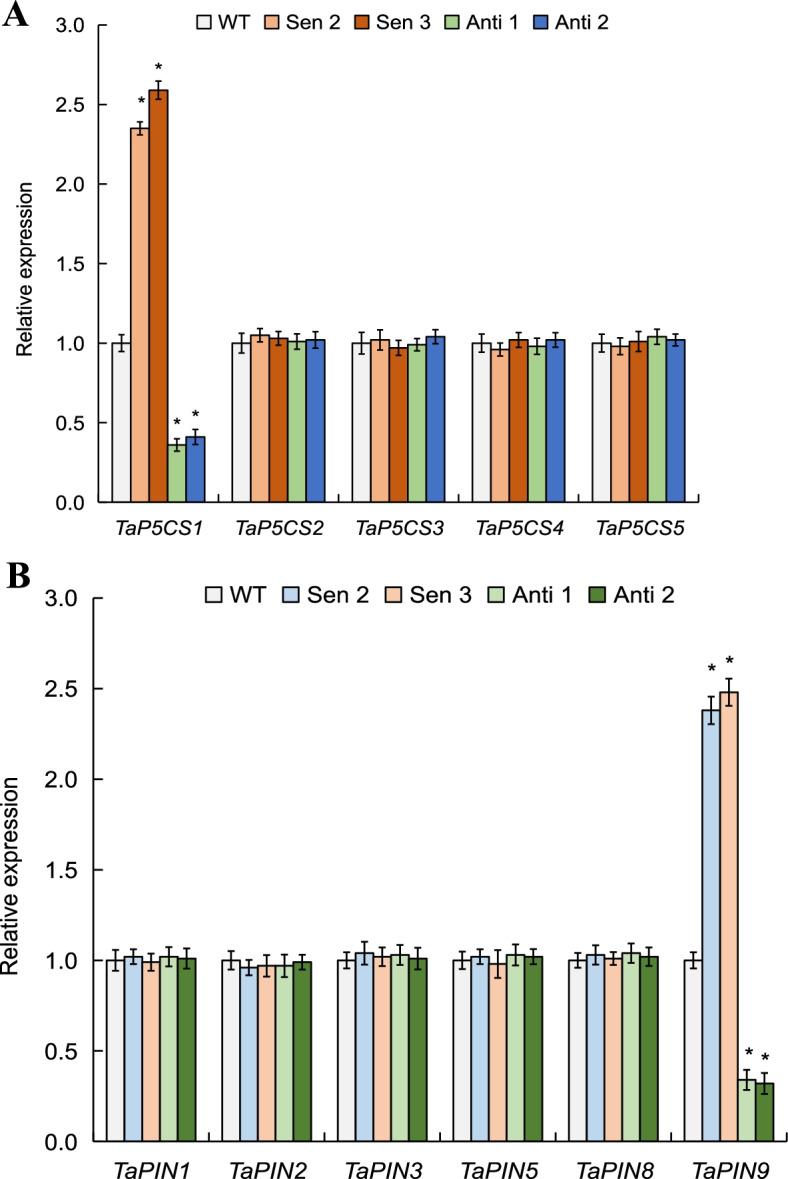
Expression patterns of the P5CS and PIN genes in *TaPYL4* transgenic lines under drought treatment. **A** expression patterns of P5CS family genes; **B** expression patterns of PIN family genes. Average values are derived from triplicate results. Error bars represent standard errors and symbol * indicates significant differences between the transgenic lines and WT calculated by one-way ANOVA with significance level of 0.05

Expression patterns of the genes in delta-1-pyrroline-5-carboxylate synthetase (P5CS) family impacting proline biosynthesis and in PIN-FORMED (PIN) family involving root system architecture (RAS) establishment were investigated, with an aim to understand the molecular processes related to the *TaPYL4*-mediated osmolyte accumulation and RSA property under drought conditions. Among five P5CS family genes examined, *TaP5CS1* was modified significantly in transcription in the drought-challenged *TaPYL4* transgenic lines, displaying higher expression levels in Sen 2 and Sen 3 whereas lower ones in Anti 1 and Anti 2 compared with the WT plants (Fig. 6A). Among six genes in the PIN family, similar to *TaP5CS1*, *TaPIN9* displayed modified transcripts in the *TaPYL4* transgenic lines with respect to WT under drought treatment, with more transcripts shown in Sen 2 and Sen 3 whereas less expression levels in Anti 1 and Anti 2 than the WT plants (Fig. 6B). These genes in P5CS and PIN families (i.e., *TaP5CS1* and *TaPIN9*) modified significantly on expression efficiency in *TaPYL4* transgenic lines under drought treatment, contrasting to other genes in P5CS and PIN families examined that unaltered transcripts abundance among the transgenic and WT plants. Therefore, it is suggested that the *TaPYL4-*improved drought adaptation is associated with the modified transcription of distinct genes in P5CS and PIN-FORMED families that regulate osmolyte accumulation and RSA establishment.

### Functions of *TaP5CS1* and *TaPIN9* in mediating plant drought response

**Fig. 7:**
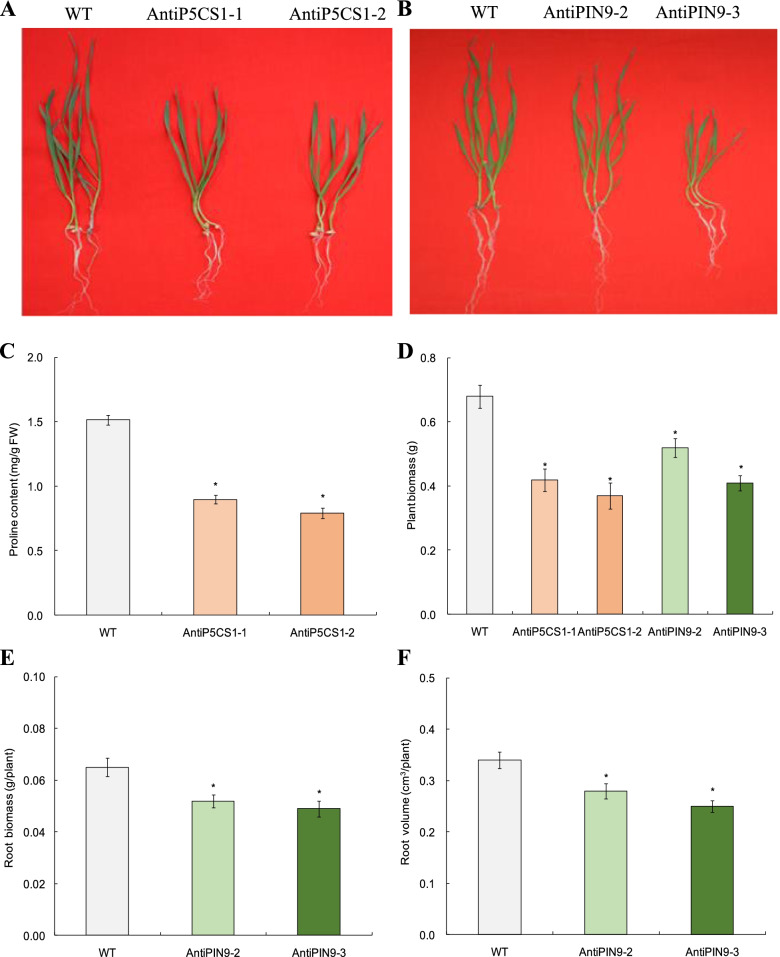
Functional characterizations of *TaP5CS1* and *TaPIN9* under drought stress treatment. **A** phenotypes of transgenic lines with knockdown expression of *TaP5CS1*; **B** phenotypes of transgenic lines with knockdown expression of *TaPIN9*; **C** proline contents; **D** plant biomass; **E** root biomass; **F** root volume. WT, wild type; AntiTaP5CS1–1 and AntiTaP5CS1–2, transgenic lines with knockdown expression of *TaP5CS1*; AntiPIN9–2 and AntiPIN9–3, transgenic lines with knockdown expression of *TaPIN9*. In **C**-**F** average values are derived from triplicate results. Error bars represent standard errors and symbol * indicates significant differences between the transgenic lines and WT calculated by one-way ANOVA with significance level of 0.05

*TaP5CS1* and *TaPIN9*, two members in P5CS and PIN families that modified transcription underlying *TaPYL4* regulation, were subjected to transgene analyses to address their functions in mediating plant drought stress response. Results indicated that the lines with significant knockdown expression of *TaP5CS1*, namely, AntiP5CS1–1 and AntiP5CS1–2 (Additional file 5), were significantly alleviated on phenotypes (Fig. 7A), proline accumulation in plants (Fig. 7B), and plant dry mass production (Fig. 7C) relative to WT under drought treatment. Likewise, the lines with drastic knockdown expression of *TaPIN9* (i.e., AntiPIN9–2 and AntiPIN9–3) (Additional file 6) displayed significant modification on RSA establishment and plant drought response. Compared with WT, AntiPIN9–2 and AntiPIN9–3 drastically alleviated root growth traits and plant biomass under drought treatment, showing smaller stature of plants (Fig. 7A), less dry mass accumulation in whole plant (Fig. 7D) and in roots (Fig. 7E), and lower root volume (Fig. 7F) than the wild type. Therefore, the transgene results on *TaP5CS1* and *TaPIN9* validated the gene functions in promoting osmolyte accumulation and in improving RSA establishment underlying *TaPYL4* regulation, respectively. Therefore, these genes act as the crucial modulators in plant adaptation to drought stress via enhancement of osmolyte-regulatory capacity and improvement of RSA establishment.

### Transcriptome profile mediated by *TaPYL4* under drought stress conditions

Transcriptome profiles in the drought-challenged *TaPYL4* transgenic line (Sen 2) and WT were investigated based on high throughput RNA-seq analyses to systematically understand the molecular processes underlying *TaPYL4* modulation. Results indicated that in total of 3850 genes, including 2613 upregulated and 1237 downregulated, were shown to be differentially expressed (DE) in the transgenic line after drought stress treatment (Additional files 7-8). To be sure of reproducibility for the transcriptome results, ten DE genes including five of upregulated whereas another five of downregulated in expression were selected and subjected to qRT-PCR analysis. As expected, all of the five genes with upregulated pattern (i.e., *TaWRK2*, *TaWRKY28*, *TaCML31*, *TaMPK18*, and *TaZFP1*) displayed higher expression levels in Sen 2 with respect to WT, with comparable folds increased as shown in RNA-seq analysis. Likewise, the five DE genes with downregulated in expression (i.e., *TaPAO*, *TaCA*, *TaCP450*, *TaUBI6*, and *TaFR1*) exhibited decreased transcripts in Sen 2 relative to WT plants under drought condition (Additional file 9). Moreover, these DE genes in Anti 1 exhibited reverse expression pattern to that shown in Sen 2 mentioned above, namely, the five upregulated DE genes were significantly decreased whereas the five downregulated DE ones were drastically elevated on expression levels compared with the WT plants (Additional file 10). These qRT-PCR results confirmed the credibility for the transcriptome profile underlying modulation of *TaPYL4* upon drought stress.

**Fig. 8:**
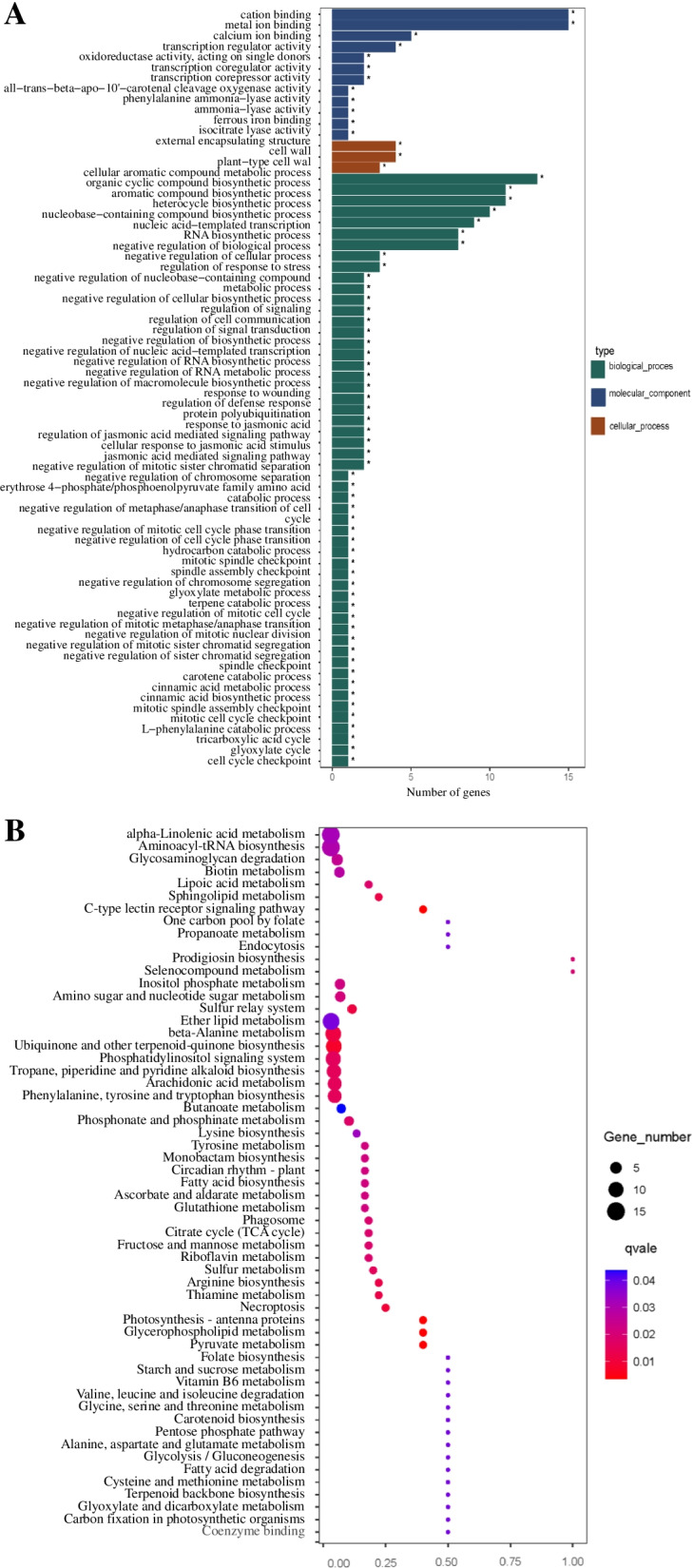
Characterization of the differentially expressed genes in the drought-challenged *TaPYL4* transgenic lines. **A** GO terms that are overrepresented by the upregulated DE genes; **B** biochemical pathways are enriched by the upregulated DEGs

Based on the gene ontology (GO) analysis on the DE genes, the DE genes were categorized into the GO terms associated with “*biological process*”, “*molecular components*”, and “*cellular process*”. Among these, the DE genes in the GO term “*biological process*” are related to 12 processes, mainly overrepresented by those of cation binding, metal ion binding, and calcium ion binding, etc.; the DE genes in GO term ‘*molecular component*’ translate three classes of proteins, including constitution of external encapsulating structure, cell wall, and plant-type cell wall; the DE genes in GO term‘*cellular process*’ associate with 54 kinds of cellular functions, mainly functioning in aromatic compound metabolic process, organic cyclic compound biosynthetic process, and aromatic compound biosynthetic process, etc. (Fig. 8A). KEGG analysis revealed that the DE genes identified in the drought-challenged *TaPYL4* overexpression lines suggests a total of 57 of biochemical pathways underlying control of *TaPYL4*, which were mainly overrepresented by the biochemical pathways associated with beta-alanine metabolism, ubiquinone and other terpenoid-quinone biosynthesis, and phosphatidylinositol signaling system, etc. (Fig. 8B). From the transcriptome analysis results, a suite of upregulated DE genes were found to be functional in biological processes related to the *TaPYL4*-mediated drought tolerance, such as stomata movement, osmolyte (i.e., proline) metabolism, and root system architecture (RSA) (i.e., auxin responsive and root development-associated) behavior. Of which, the DE genes functional in stomata include transmembrane ATPase for substance translocation (TraesCS4D02G339000) and PXG (TraesCS2D02G364500); the DE genes associated with proline metabolism include proline-rich nuclear receptor coactivator (TraesCS5B02G261200, TraesCS5A02G262800), arginine and proline metabolism (TraesCS2A02G548100, TraesCS2D02G549200, TraesCS2A02G334600), and proline dehydrogenase 2 (TraesCS1D02G212400, TraesCS1A02G209100, TraesCS1B02G223300; the DE genes involved in RSA establishment include auxin responsive protein (TraesCS4B02G287100, TraesCS2A02G183900, TraesCS3A02G331900), auxin-responsive protein IAA9 (TraesCS6A02G373300), auxin response factor 1 (TraesCS3D02G166700), auxin-responsive SAUR family member (TraesCS7B02G370600), auxin-induced in root culture (TraesCS7B02G152200), and auxin response factor 12 (TraesCS2D02G548900) (Additional file 7). These results from the transcriptome profiles mediated by *TaPYL4* suggested that this ABR gene globally modulates the transcription of quantities of genes, whose modified expression levels lead to plant drought response through regulating diverse stress responsive-associated physiological processes and biochemical pathways, such as those related to stomata movement, osmolytes biosynthesis, and RSA establishment.

## Discussion

As an essential phytohormone in plant species, abscisic acid (ABA) regulates diverse biological processes associated with plant growth and development as well as plant responses to a large set of environmental stresses, including osmotic stressors [12]. Thus far, genetic and biochemical analyses have identified a large set of ABR (PYR/PYL) family members across various plant species, especially the model plant Arabidopsis [23]. Moreover, a subset of the PYL proteins acting as key regulators in ABA signaling have been functionally characterized [24]. In this study, we performed characterization analysis on *TaPYL4* and revealed that it shares high similarities to its PYL counterparts distributed in diverse plant species. TaPYL4 also harbors nine conserved domains (CL1 to CL9) involving binding ABA and interacting with its partners (i.e., PP2Cs). Therefore, *TaPYL4* acts as one of the PYL family members in *T. aestivum*. Our experimental analysis in determination of the subcellular localization of TaPYL4 indicated its position onto plasma membrane and nucleus after ER assortment, suggesting where it percepts the ABA signaling and exerts biological roles.

Transcripts of the genes in PYL family were detected in various plant species, such as rice [25], tomato [26, 27], cotton [18], and the rubber tree [28]. However, although the expression of PYL genes is shown in various tissues, such as seeds, tissues root and leaf, and organs flower and fruit, much more of the *PYL* transcripts are specified in distinct organs/tissues. For example, rice *OsPYL7* and *OsPYL8* are abundantly expressed in embryos, *OsPYL*3 and *OsPYL*5 primarily express in leaves, while *OsPYL1* predominantly expresses in roots [29]. Moreover, upon osmotic stress, a subset of *PYR/PYL/RCAR* genes induces expression levels due to enhanced cellular ABA levels in plants [30, 31]. In this study, we evaluated the *TaPYL4* expression patterns in root and aerial tissues upon drought stress. *TaPYL4* transcripts in tissues were enhanced by drought stressor and its upregulated expression initiated by drought was restored to low levels following the progression of normal recovery condition. These expression results suggested the sensitive response of *TaPYL4* to external drought signaling. Thus far, characterization on *cis*-acting regulatory elements in the promoters of drought stress-responsive genes has indicated that ABRE, which is specified by motifs ACGTG, AACCCGG, and CGTACGTGCA, frequently situates in the promoter region and regulates gene transcription in responding to osmotic stresses. This element leads to promoted gene transcription efficiencies by interacting with DNA binding domains harboring in proteins of the bZIP transcription factors [32]. Therefore, *TaPYL4* modified expression upon drought stress associates distinct *cis*-acting regulatory elements in its promoter, such as ABRE or other uncharacterized ones. By searching *TaPYL4* promoters using online tool PLACE, we identified a set of elements involving stress and ABA responses, such as ABRE, which are situated at − 1702 (sequence ACGTG), 1229 (AACCCGG), 1676 (CGTACGTGCA) and 1679 (ACGTG) (Additional file 11). These elements are therefore suggested to be involved in the *TaPYL4* response to drought stress at transcriptional level. Further characterizing these elements can provide insights into the transcription mechanisms of the PYL family members in response to drought signaling.

The PYL family members mediate extensively plant growth and osmotic stress responses. For example, transgenic lines overexpressing rice *OsPYL/RCAR5* are hypersensitive to ABA during seedling stage [33]. In contrast, PYL genes *OsPYL/RCAR5*, *OsPYL3*, and *OsPYL9* confer plants improved drought tolerance [34]. Elevation of AtPYL5 and AtPYL9 abundance enhances plant ABA responses and drought tolerance through interacting with a set of clade A PP2C proteins [17]. Likewise, several other members in ABA receptor family, such as Arabidopsis *RCAR11-RCAR14* and rice *OsPYL6* and *OsPYL10*, has been conformed to play critical roles in enhancing plant drought tolerance by regulating transcription of a subset of genes associated with stress response or defensiveness in the ABA-dependent pathways [10, 20, 21]. These findings together suggested that the PYL genes act as crucial modulators in plant adaptation to drought stress. In this study, we used transgene analysis to characterize the function of *TaPYL4* in mediating plant drought response. The lines with *TaPYL4* overexpression (i.e., Sen 2 and Sen 3) drastically improved plant growth, RSA establishment, and biomass production whereas those with target knockdown (i.e., Anti 1 and Anti 2) alleviated significantly on above growth traits with respect to wild type under drought treatment. These transgene results confirmed the essential role of *TaPYL4* in regulating plant drought tolerance. Additionally, we performed a field experiment for the *TaPYL4* transgenic lines (Sen 2, Sen 3, Anti 1, and Anti 2) and WT under regularly normal irrigation (irrigated at spring stages of jointing and mid-filling) and water-saving (irrigated at spring stage of jointing) conditions. Analysis on yields indicated that overexpression and knockdown expression of *TaPYL4* leads to increased and decreased yields, respectively compared with WT under water-saving cultivation condition. Under normal irrigation condition, no penalties on yields were found in the *TaPYL4* transgenic lines relative to WT plants (Additional file 12). These results suggest the potential value of *TaPYL4* in molecular breeding the drought-tolerant cultivars in *T. aestivum*.

Stomata movement acts as one effective mechanism for plant response to osmotic stresses via an ABA-dependent pathway. Enhanced capacity of plants for water maintenance under drought condition associates promoted stomatal closure rate (SCR) initiated by induced ABA amounts, given the mediation of ion transport systems situated in plasma membrane of guard cells that modify turgor and volume of cells [35]. In this study, we investigated the stomata closing nature upon drought signaling underlying *TaPYL4* regulation. Results indicated significantly modified stomata closing rate (SCR) in *TaPYL4* transgenic lines compared with WT. The SCR behaviors in Sen 2 and Sen 3 were promoted whereas Anti 1 and Anti 2 slowed within a 1 h-regime of drought duration. These SCR analysis results were in concordance with the positive role of *TaPYL4* in regulating water retention capacity of the drought-treated plants. Previously, anion channels in guard cells, such as those categorized into S-type and R-type, were documented to be activated upon osmotic stresses, impacting stomata movement and SCR by mediating anion efflux from guard cells, depolarize the potassium efflux channels situated in plasma membrane, and modify guard cell osmotic potential [36]. Further characterizing the mechanisms underlying *TaPTL4-*mediated anion efflux in guard cells can deepen understanding the stomata movement mediated by ABRs in *T. aestivum*. Another issue should be mentioned although the lines overexpressing *TaPYL4* (i.e., Sen 2 and Sen 3) were much faster on stomatal closing upon drought signaling than wild type in our investigation, they also possessed increased photosynthetic rate (Pn). We speculate that the improved photosynthesis mediated by *TaPYL4* under drought condition is largely ascribed to its improved photosynthetic system activation (i.e, improved PSII and lowered NPQ) that compensates its adverse effects of promoting stomata movement on Pn behavior. Similar finding has been documented on *PeCHYR1*, a ubiquitin E3 ligase gene in *P. euphratica*, whose up-regulation confers plants promoted stomatal closure together with enhanced photosynthetic activity and biomass accumulation [37]. Further investigation on the relation between stomata closure and photosynthetic function mediated by *TaPYL4* can provide insights into plant drought adaptation for cereal crops.

Distinct PYL members consist of core ABA signaling modules with PP2C and SnRK2 proteins to be functional in plant osmotic stress responses [38, 39]. Once interacted by ABRs upon osmotic stressor, PP2C proteins lead to released inhibition on the activity of SNF1-related protein kinase 2 (SnRK2) proteins [16], which activate the downstream regulators in ABA-dependent pathway, such as transcription factors, S-type anion channel SLAC1, and a large set of proteins functional in various groups thorough phosphorylation mechanism. These processes thereby lead to drought-acclimated stomata movement and plant stress responses [40]. In this study, we performed yeast two-hybridization assays to identify the core signaling module components aside from TaPYL4. Our results indicated that TaPP2C2, a clade A PP2C member and TaSnRK2.1, a member of the SnRK2 family, constitute a core ABA signaling module, namely, TaPYL4/TaPP2C2/TaSnRK2.1, given their specific protein-protein interactions. In this regard, it is concluded that the TaPYL4-mediated plant drought response is accomplished via the ABA dependent pathways that initiate plant drought stress responses. Further functional characterization of TaPP2C2 and TaSnRK2.1 can benefit understanding of the molecular mechanisms underlying the ABA signal transduction pathways in *T. aestivum*.

Plant accumulation of osmolytes under osmotic stresses decreases cellular osmotic potential, enhancing cell water retention capacity and alleviating injury extent of plants initiated by stressors [41]. Proline and soluble sugar are two types of crucial osmolytes, whose induction under drought stress contribute largely to plant drought adaptation [42]. In this study, we investigated the *TaPYL4*-mediated modification on above two osmolytes by assessing contents of them in drought-challenged transgenic lines. As expected, we found that *TaPYL4* positively regulates proline and soluble sugar accumulation; higher contents of osmolytes in Sen 2 and Sen 3 whereas lower contents of those were observed in Anti 1 and Anti 2 compared with wild type. These results suggested that *TaPYL4* enhances osmolytes accumulation in plants once challenged by drought stress. Based on expression analysis, we found the transcripts of *TaP5CS1*, a member of Δ1-pyrroline-5-carboxylate synthetase (P5CS) family acting as a rate-limiting regulator in proline biosynthesis [7, 43], significantly modified expression in transgenic lines under drought treatment. Our online search analysis revealed that the ABA responsiveness motif ABRE (ACGTG, − 1274, − 529, 225, 406, and 1401) and that the drought-inducible element MBS (CAACTG, − 1157 and − 313) frequently situate in *TaP5CS1* promoter, being consistent with its upregulated expression upon drought stress. Further transgene analysis on *TaP5CS1* validated its positive role in regulating proline accumulation under drought conditions. To date, a subset of drought-responsive proteins including those involving osmoprotectant biosynthesis metabolisms has been documented [7]. Further addressing the transcriptional mechanisms of *TaP5CS1* mediated by *TaPYL4* can help elucidate the osmotic-regulatory mechanism underlying PYL members in drought response in *T. aestivum*.

Auxin modulates widely plant physiological processes associated with morphogenesis, organogenesis, apical dominance, embryo formation, vascular differentiation, and stress responses [27]. The subcellular localization of auxin possesses polar nature in root tissue, which is mediated largely by the PIN-FORMED (PIN) proteins given their roles in determining directional flow and polar transport of auxin at cellular level [44]. Distinct PIN proteins also mediate polar re-allocation of auxin from tissue shoot to root tip [45], by which to regulate lateral root differentiation [46] and root system architecture (RSA) establishment [47]. In this study, we addressed the issue whether *TaPYL4*-mediated RSA establishment to contribute to plant drought tolerance by investigating the expression patterns of the PIN family genes in drought-challenged *TaPYL4* transgenic lines. We found that *TaPIN9* is modified on transcription, with more transcripts in Sen 2 and Sen 3 and less ones in Anti 1 and Anti 2 than WT plants. Online search analysis revealed that the elements associated with drought response, such as ABRE (− 241, − 630, − 1682, and 1770) and MBS (− 1472 and 942), are situated in *TaPIN9* promoter region, suggesting the involvement of them in mediating gene transcription under drought stress condition. Further tangsgene analysis confirmed the positive function of *TaPIN9* in regulating RSA establishment and plant drymass production under drought treatment. Although it was found that the *TaPIN9* transcripts are upregulated in the drought-challenged *TaPYL4* overexpressing lines (Sen2 and Sen 3), our yeast two-hybrid assay did not detect the interactions between TaPYL4 and TaPIN9, as that shown between the former and TaP5CS1 (Additional file 13). Therefore, our investigation suggested that *TaPYL4* modulates RSA establishment by transcriptionally regulating *TaPIN9* through an indirect manner mediated by distinct uncharacterized mediators, such as transcription factor(s) that is regulated underlying the wheat PYL member. Previously, the clade A PP2A phosphatases were documented to mediate cellular auxin transport through a reversible phosphorylation mechanism [48]. This finding suggests the putative functional pathways constituted by PYL/PP2C/PIN to regulate auxin transport at cellular level and to impact on the RSA behavior of plants challenged by drought stress.

**Fig. 9:**
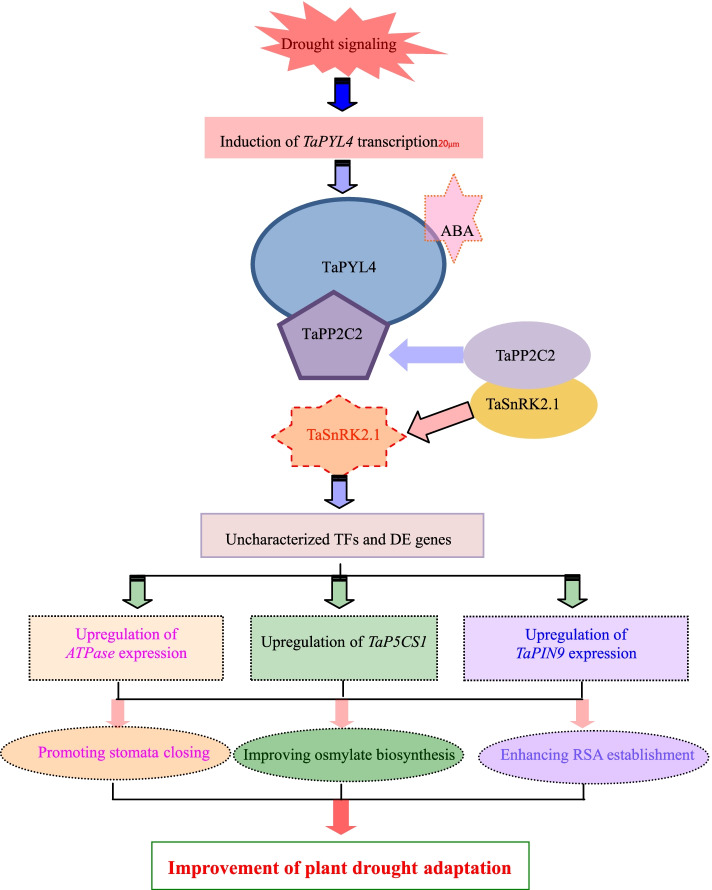
Putative diagram for biological pathways associated with plant drought response underlying modulation of *TaPYL4*

ABA signaling initiated by osmotic stressors modifies a quantity of physiological and biochemical processes which are corporately integrated in plant drought responses [11, 49]. In this study, we performed high throughput RNA-seq analyses to identify the *TaPYL4*-mediated transcriptome profile under drought stress condition. We identified a large set of differentially expressed (DE) genes in Sen 2 compared with wild type after drought treatment. These DE genes are enriched into the GO terms associated with “*biological process*”, “*molecular components*”, and “*cellular process*” and overrepresented by KEGG pathways related to diverse biochemical pathway, suggesting the complex nature in *TaPYL4*-mediated plant drought response. Based on gene expression analysis and the GO terms and KEGG pathways enriched by the DE genes, we put forth the working model of *TaPYL4* in regulating plant drought responses (Fig. 9). Upon plant perception of drought signaling, the *TaPYL4* transcripts are upregulated across roots and aerial tissues. The enhanced translation of the TaPYL4 protein interacts with TaPP2C2, a member of the clade A PP2C family, releasing the activation of the kinase TaSnRK2.1 by which to elevate the transcript abundance for genes involving regulation of stomata movement, osmolytes accumulation (i.e., *TaP5CS1*), and RSA establishment (i.e., *TaPIN9*), and stress defensiveness. These genes then function in a synergic manner to contribute to the plant drought tolerance.

## Conclusions

Wheat ABR gene *TaPYL4* shares high similarities to its homologous genes across various plant species. The expression levels of *TaPYL4* in root and aerial tissues are sensitive in response to drought stress. TaPYL4 targets onto plasma membrane and nucleus where it involves establishment of a putative core ABA signaling module TaPYL4/TaPP2C2/TaSnRK2.1. Overexpression of *TaPYL4* confers plants significantly improved drought tolerance, due to gene function in positively regulating stomata movement, osmolytes biosynthesis, and RSA establishment. *TaP5CS1*, a gene in the P5CS family and *TaPIN9*, a member of the PIN-FORMED family, are transcriptionally regulated by *TaPYL4*, contributing to plant drought adaptation by promoting proline accumulation and RSA formation, respectively. *TaPYL4* is also involved in the regulation of transcription efficiencies of numerous genes, which are functional in various biological processes associated with plant drought stress defensiveness mainly through the stress defensive pathway.

## Methods

All methods were carried out in accordance with relevant guidelines.

### Characterization analysis on *TaPYL4*

*TaPYL4* (GenBank accession No. XM_044507276) was significantly upregulated in expression upon drought stress in our previous RNA-seq analysis (unpublished data). This finding prompted us to characterize it in mediating plant drought response in more detail. This ABR gene has three homeologs across chromosomes 4A (*TaPYL4-4A*, TraesCS4A02G114400), 4B (*TaPYL4-4B*, TraesCS4B02G189800), and 4D (*TaPYL4-4D*, TraesCS4D02G191200), which share high similarities at nucleic acid and amino acid levels each other (98.5–99.2%, Supplemental files 14A and 14B). Of which, *TaPYL4-4A* displayed strongly induced transcripts upon drought stress relative to other homeologs (Additional file 14C). Therefore, we concentrated on characterization of *TaPYL4-4A* in this investigation (simplified by *TaPYL4* hereafter). The conserved domains harbored in the TaPYL4 protein were defined base on previous literature [38]. The homologous genes of *TaPYL4* in plant species were identified by sequence search analysis against the GenBank database of National Center in Biotechnology Information (NCBI). Phylogenetic relations among *TaPYL4* and its plant counterparts were established using the MegAlign algorithm supplemented in the DNAStar software.

### Determination of TaPYL4 subcellular localization

The subcellular localization of TaPYL4 was defined based on detecting the signals of green fluorescent protein (GFP) initiated by fusion TaPYL4-GFP at cellular level. With this purpose, the open reading frame (ORF) of *TaPYL4* was amplified using RT-PCR together with gene specific primers (Additional file 15). It was then integrated into the binary vector pCAMBIA3300 in frame with the reporter gene *GFP* downstream of the CaMV35S promoter. The expression cassette harboring *TaPYL4-GFP* was subjected to genetic transformation onto *A. tumefaciens* strain (EHA105) following the conventional heat-shock transformation approach, and then transformed epidermal cells of *N. benthamiana* through an *A. tumefaciens*-mediated transformation method. The GFP signals derived from the epidermal cells transformed with *TaPYL4-GFP* and those from control harboring empty vector (with *GFP* as reporter gene) were observed under fluorescent microscope after 48 h of infiltration as described previously [50]. Additionally, we used protoplast expression system of *N. benthamiana* to define the location of TaPYL4 at cellular level. With this aim, young leaves *N. benthamiana* were obtained following the procedure of previously described [51]. Transformation on isolated protoplasts using the cassettes harboring TaPYL4-GFP was conducted following a modified PEG-mediated protocol [52]. The signals in protoplasts initiated by the fusion were observed under fluorescent microscope after 24 h of infiltration with the *A. tumefaciens* transformants.

### Yeast two-hybrid assays

Yeast two-hybrid assays were performed to identify the partner interacting with TaPYL4 as well as that involving interaction with the latter, with an aim to identify the putative core ABA signaling module covering TaPYL4. In this context, six genes in the PP2C family and seven ones in the SnRK2 family in *T. aestivum* were identified from the GenBank database in NCBI. The members of PP2C family subjected to assays included *TaPP2C1–1A*, *TaPP2C2-1A*, *TaPP2C3-1B*, *TaPP2C4-1D*, *TaPP2C5-1A*, and *TaPP2C6-3B* (simplified by *TaPP2C1* to *TaPP2C6*). The genes of SnRK2 family subjected to assays contained *TaSnRK2.1–2A*, *TaSnRK2.2-2A*, *TaSnRK2.3-1A*, *TaSnRK2.4-3A*, *TaSnRK2.5-2A*, *TaSnRK2.6-2A*, and *TaSnRK2.7-1A* (simplified by *TaSnRK2.1*-*TaSnRK2.7*). Information of the genes in PP2C and SnRK2 families subjected to yeast two-hybrid assays is shown in Additional file 15. During assays, TaPYL4 was firstly used as a bait whereas the proteins in PP2C family as preys separately to identify target PP2C partners interacted by the PYL member. During co-cultivation for the yeast transformants expressing *TaPYL4* and each *TaPP2C* member in host strain (AH109), exogenous abscisic acid (ABA) was supplemented in the selecting solid medium with concentration of 1 μM to elicit the putative protein interaction process. Likewise, to determine the SnRK2 members involving the constitution of core ABA signaling module, the PP2C member identified was used as bait whereas the proteins in SnRK2 family as preys separately. Procedures for these yeast two-hybrid assays were performed following the manufacturer’s instructions [53]. Gene specific primers used for the amplification of *TaPYL4* and the genes in PP2C and SnRK2 families mentioned are shown in Additional file 15.

### Expression analysis of *TaPYL4* upon drought stress

The expression patterns of *TaPYL4* upon drought stress were evaluated using Shimai 22, a cultivar to be drought-tolerant (kindly provided for research purpose by the Wheat Breeding Institute, Shijiazhuang Research Academy of Agriculture and Forestry). Briefly, the seeds were regularly germinated in a growth chamber and the young seedlings were cultured in a standard MS solution as described previously [54]. At the second-leaf stage, they were subjected to simulated drought treatment by growing in a modified MS solution containing different concentrations of polyethylene glycol 6000 (PEG-6000), namely, 0, 1, 5, 10, and 15% (w/v) which correspond to osmotic potentials of 0, − 0.35, − 0.81, − 1.08, and − 1.27 mPa, respectively. The roots and leaves of seedlings were sampled after 27 h of the treatments. To understand the temporal effects of drought stressor on gene expression, tissues mentioned above were collected at 1, 3, 9 and 27 h after drought stress treatment (10% of PEG). Additionally, aliquots of seedlings treated by 27 h of 10% PEG were re-subjected to regular growth condition by growing in standard MS solution to define the gene response to normally recovered conditions. With this purpose, tissues mentioned were sampled at 1, 3, 9, and 27 h following the recovery treatment. Transcripts of the target gene in collected samples were evaluated based on qRT-PCR performed as described previously [50], using gene specific primers (Additional file 15). A constitutive gene *Tatubulin* in *T. aestivum* was used as an internal reference to normalize the target transcripts. To avoid the effects of circadian rhythm or mechanical stimuli on gene expression, the tissues subjected to evaluation of target gene transcripts were all sampled during light phase under photoperiod of 16 h (light)/8 h (dark) and the wheat seedlings were gently transferred across culture solutions.

### Assays for growth traits of *TaPYL4* transgenic lines

A suite of transgenic lines with modified expression of *TaPYL4* were generated to characterize the target gene function in mediating plant drought response. With this aim, the ORF of *TaPYL4* was amplified in both sense and anti-sense orientations based on RT-PCR using gene specific primers (Additional file 15). They were then separately inserted into the *Nco*I/*BastE*II sites in binary vector pCAMBIA3301 under control of the CaMV35S promoter. The expression cassettes harboring target gene were subjected to transformation onto *A. tumefaciens* (strain EHA105) using the conventional heat-shock approach, which were further subjected to genetic transformation onto *T. aestivum* (cv. Shimai 22) based on an *A. tumefaciens*–mediated transformation method as described previously [50]. The expression levels of target gene in transgenic lines, namely, six lines with target overexpression (Sen 1 to Sen 6) and five ones with knockdown expression of the target gene (Anti 1 to Anti 5), were evaluated based on qRT-PCR using gene specific primers (Additional file 15).

Sen 2 and Sen 3, two lines at T3 generation with more target transcripts and Anti 1 and Anti 2, two lines with lowered *TaPYL4* expression with one copy insertion each (Additional file 3), were selected to address the gene function in mediating plant drought response. To this end, these transgenic lines together with wild type (WT) were cultured in pots filled by mixed soil (half vermiculate and another half fertile soil) under normal growth condition (supplied daily by tap water to sustain 70–75% relative soil moisture) to the second-leaf stage. Afterwards, they were then subjected to drought treatment by sustaining 55–60% of relative soil moisture, with water amount applied daily monitored by soil water potential meter (TRS-IIN, China). Three weeks after the treatments, the growth traits in transgenic lines including phenotypes, biomass in aerial tissues and roots, and root volumes were assessed. Of which, phenotypes were shown as images taken by a digital camera; biomass of plant tissues and root volumes mentioned was obtained following the conventional assay approach.

### Assays of drought stress-associated physiological traits and photosynthetic parameters

Drought response-associated traits and photosynthetic parameters were evaluated in the transgenic lines after drought treatment. The drought response-associated traits assessed included stomata closing rate (SCR), leaf water losing rate (WLR), and osmolytes contents. Of which, SCR was determined based on recordation of stomata aperture at indicated time points (i.e., 0, 0.5, 1, and 2 h) following the drought treatment as previously [55]; WLR values were calculated according to the decreased fresh weights in detached leaves at indicated time points (0.25, 0.5, 1, 2, and 3 h) following drought stressor with respect to that at the time point 0 h (prior to treatment) as described by Imadi et al. (2015) [56]; the contents of osmolytes, including proline and soluble sugar, were assessed according to the methods reported by Liu et al. (2020) [57]. The photosynthetic parameters assayed in the drought-challenged transgenic lines included photosynthetic rate (Pn), stomatal conductance (gs), photosystem II photochemical efficiency (ΦPSII), and non-photochemical quenching coefficient (NPQ). Of which, Pn and gs were measured in the upper fully expanded leaves using a photosynthesis system (LiCOR-6200) following the manufacturers’ instructions; ΦPSII and NPQ in the representative leaf samples were evaluated as reported previously [50].

### Analyses of expression and function of P5CS and PIN-FORMED family genes

To characterize the molecular processes associated with the improved osmolyte biosynthesis and the root system architecture (RSA) establishment underlying *TaPYL4* modulation, five genes in delta-1-pyrroline-5-carboxylate synthetase (P5CS) family impacting on proline biosynthesis in *T. aestivum*, including *TaP5CS1* to *TaP5CS5*, were identified in the NCBI GenBank database. Likewise, six genes in PIN-FORMED (PIN) family of wheat, including *TaPIN1* to *TaPIN3*, *TaPIN5*, *TaPIN8*, and *TaPIN9*, were obtained. Information of the genes in P5CS and PIN families mentioned are shown in Additional file 15. The transcripts of above P5CS and PIN family genes in the transgenic lines and WT after drought treatment were evaluated based on qRT-PCR using gene primer pairs (Additional file 15).

*TaP5CS1* and *TaPIN9*, two genes showing significantly more transcripts in Sen 2 and Sen 3 whereas less ones in Anti 1 and Anti 2 compared with wild type, were subjected to functional characterizations based on transgene analysis. With this purpose, the ORFs of *TaP5CS1* and *TaPIN9* were amplified in anti-sense orientation based on RT-PCR. They were then integrated separately into the *Nco*I/*BstE*II sites in the binary vector pCAMBIA3301 under control of the CaMV35 promoter. Genetic transformation for these genes onto *A. tumefaciens* strain EHA105 and further genetic transformation of them onto *T. aestivum* (*cv*. Shimai 22) were performed similarly in generation of the knockdown expression lines of *TaPYL4* mentioned above. Two lines with significantly downregulated *TaP5CS1* expression, namely, AntiP5CS1–1 and AntiP5CS1–2 (Additional file 15) and two ones with lowered *TaPIN9* transcription, AntiPIN9–2 and AntiPIN9–3 (Additional file 15), were subjected to drought treatment as mentioned previously. To this end, the knockdown lines mentioned and the WT plants were cultured under normal growth condition to the second-leaf stage and then subjected to 3 weeks of drought treatment as aforementioned. After that, proline contents and biomass in lines AntiP5CS1–1 and AntiP5CS1–2 were assessed. Likewise, biomass in aerial tissues and roots and root volumes in lines AntiPIN9–2 and AntiPIN9–3 were analyzed after drought treatment. The procedures for assessing above traits were similar to those conducted in the drought-challenged *TaPYL4* transgenic lines.

### Transcriptome analysis

To characterize the transcriptome profile modulated by *TaPYL4* under drought condition, Sen 2 that significantly upregulated expression of *TaPYL4* together with WT was subjected to high throughput RNA-seq analyses after drought treatment. Briefly, total RNA in the roots of Sen 2 and WT was separately extracted using the TRIzol reagent (Invitrogen). They were then used to construct the strand-specific RNA-seq libraries in triplicates, following the approach described previously [58]. The transcripts in the libraries were sequenced using an Illumina HiSeq 2500 sequencing platform. Raw reads generated from the libraries were further processed by removing the adaptors in reads and those to be low-quality using the tool referred to as Trimmomatic [59]. The resulting ones generated were then subjected to an alignment analysis by searching against the *T. aestivum* reference transcript database (Novogene Co, LTd, Beijing). Differentially expressed (DE) genes in Sen 2 after drought treatment were defined for those with modified transcripts over 2-fold variations in the transgenic line compared with WT, using the edger program that is effective in accurately calculating the raw count data in libraries [59]. During which, the raw *P* values were defined using a false discovery rate (FDR) less than 0.05 [60]. An online tool referred to as Plant MetGenMap (http://bioinfo.bti.cornell.edu/cgi-bin/MetGenMAP/home.cgi) was adopted to characterize the KEGG pathways of the DE genes identified, using the pearl module CPAN as described previously [61].

To validate the results derived from transcriptome analysis, we randomly selected ten DE genes, including five with upregulated expression pattern whereas another five to be downregulated in expression to be subjected to qRT-PCR analysis, using same aliquots of root tissues of lines Sen 2 and Anti 1 after drought treatment as samples. The five genes with upregulated pattern for RNA-seq analysis included *TaWRK2*, wall-associated receptor kinase 2 (TraesCS3B02G007300); *TaWRKY28*, transcription factor WRKY28 (TraesCS7B02G418400); *TaCML31*, calcium-binding protein CML31 (TraesCS3B02G553900); *TaMPK18*, mitogen-activated protein kinase kinase kinase 18 (TraesCS3B02G288100); and *TaZFP1*, zinc finger protein 1 (TraesCS5A02G401200). The five DE genes shown downregulated in expression for analysis included *TaPAO*, primary amine oxidase-like (TraesCS4A02G020900); *TaCA*, carbonic anhydrase (TraesCS3A02G230000); *TaCP450*, cytochrome P450 (TraesCS7D02G271100); *TaUBI6*, putative E3 ubiquitin-protein ligase SINA-like 6 (TraesCS3B02G288100); and *TaFR1*, fatty acyl-CoA reductase 1-like (TraesCS3B02G016500). qRT-PCR was performed similarly in detecting the transcripts of *TaPYL4* as aforementioned, using gene specific primers (Additional file 15). The constitutive gene *Tatubulin* was used as internal standard to normalize target gene transcripts.

### Statistical analysis

Averages of gene transcripts, growth traits, plant biomass, stress-associated physiological traits, and the photosynthetic parameters were all derived from the triplicate results. Standard errors for the averages and statistical significance analyses for the traits assessed were determined based on the Statistical Analysis System software (SAS Corporation, Cory, NC, USA).

## Supplementary Information

**Additional file 1. **Phylogenetic relations among *TaPYL4* and its homologous genes distributed in various plant species.

**Additional file 2. **The signals initiated by TaPYL4-GFP in protoplast expression system of *N. benthamiana observed under florescent microscope.*

**Additional file 3. **Expression levels and insertion copies of the target gene in *TaPYL4* transgenic lines.

**Additional file 4. **Stomata characterization on *TaPYL4* transgenic lines of Sen 3 and Anti 2 upon drought stress.

**Additional file 5. **Expression levels of the target gene detected in transgenic lines with *TaP5CS1* knockdown expression.

**Additional file 6. **Expression levels of the target gene detected in transgenic lines with *TaPIN9* knockdown expression.

**Additional file 7.** Up-regulated differentially expressed (DE) genes underlying modulation of TaPYL4.

**Additional file 8.** Down-regulated differentially expressed (DE) genes underlying modulation of TaPYL4.

**Additional file 9.** qRT-PCR results in roots for the differentially expressed genes with upregulated expression pattern identified based on RNA-seq analysis.

**Additional file 10.** qRT-PCR results in roots for the differentially expressed genes with downregulated expression pattern identified based on RNA-seq analysis.

**Additional file 11. **The *cis*-acting regulatory element ABRE identified in the *TaPYL4* promoter region.

**Additional file 12. **The yields of transgenic lines with overexpression of knockdown expression of *TaPYL4* under normal irrigation and water-saving treatment in field experiment.

**Additional file 13.** Yeast two-hybrid assay results among TaPYL4, TaP5CS1 and TaPIN9 proteins.

**Additional file 14 **Alignment results among *TaPYL4* and its homeologs as well as expression of *TaPYL4* homeologs upon drought stress.

**Additional file 15.** PCR primers used in this study.

## Data Availability

The transcriptome sequencing data reported in this paper have been deposited in the National Center for Biotechnology Information (NCBI) database under project number PRJNA815379 and the SRA ID with number SRR18310810.
