# Supplementary material for: TaPYL4, an ABA receptor gene of wheat, positively regulates plant drought adaptation through modulating the osmotic stress-associated processes
Source: BMC Plant Biol. 2022 Sep 1;22:423. doi: 10.1186/s12870-022-03799-z (PMC9434867; doi:10.1186/s12870-022-03799-z)
Supplement: Supplementary file 1 — Additional file 1. Phylogenetic relations among TaPYL4 and its homologous genes distributed in various plant species. [file 12870_2022_3799_MOESM1_ESM.docx]

Identity (%)

*D. exilis* cDNA (LR792837)

*D. exilis* cDNA (LR761622)

*D. exilis* cDNA (LR761621)

*S. viridis* cDNA (CP050803)

*S. viridisPYL4* (MG766908)

*Z. mays* PYL5 (KJ855100)

*O. sativa* cDNA (AP014959)

*O. sativaRP Bio-226* (CP012611)

*Z. maysPYL4* (NM 001319727)

*Z. maysPYL7* (KJ855102)

*Z. japonicaPYL3* (KY475605)

*F. arundinaceaPYL4* (MN259578)

*F. elataPYL3* (KY475599)

***TaPYL4* (XM 044507276)**

*T. aestivumABAR* 7 (MG273657)

*H. vulgare* cDNA (AK361631)

*O. sativa ABAR6* (KF958293)

*P. edulis* cDNA (FP100262)

*P. edulis* cDNA（FP092179）

*H. vulgare* cDNA (AK376521)

*T. aestivum* cDNA (AK452881)

*T. aestivum* cDNA (AK335719)

M. acuminata *cDNA*(HG996468.1)

T.turgidum*PYL5* (MN416490.1)


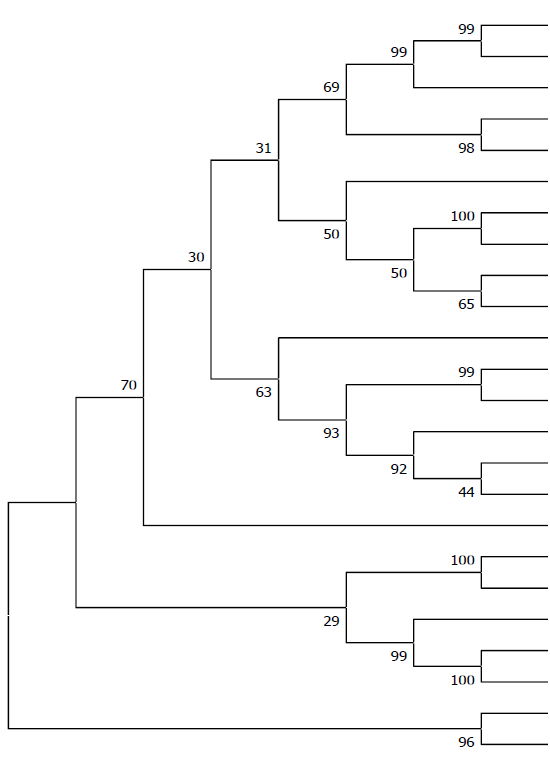


85.59

85.59

85.59

90.88

90.88

90.84

91.13

90.57

90.84

90.84

91.42

93.89

92.63

**100**

97.96

96.93

90.57

87.33

87.33

85.40

84.70

84.70

81.80

88.00

**Additional file 1** Phylogenetic relations among *TaPYL4* and its homologous genes distributed in various plant species
