# Supplementary material for: TaPYL4, an ABA receptor gene of wheat, positively regulates plant drought adaptation through modulating the osmotic stress-associated processes
Source: BMC Plant Biol. 2022 Sep 1;22:423. doi: 10.1186/s12870-022-03799-z (PMC9434867; doi:10.1186/s12870-022-03799-z)
Supplement: Supplementary file 2 — Additional file 2. The signals initiated by TaPYL4-GFP in protoplast expression system of N. benthamiana observed under florescent microscope. [file 12870_2022_3799_MOESM2_ESM.docx]

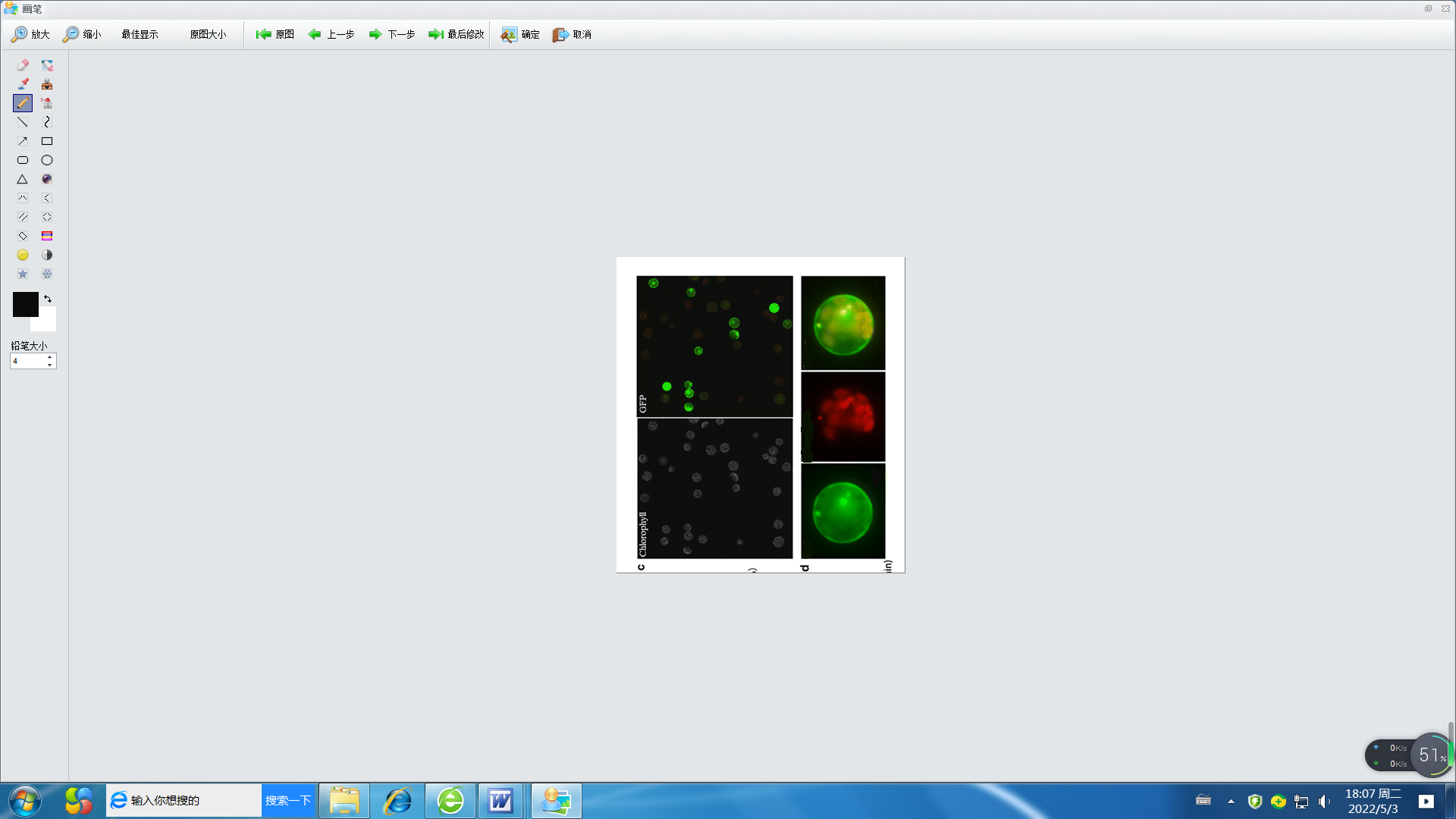

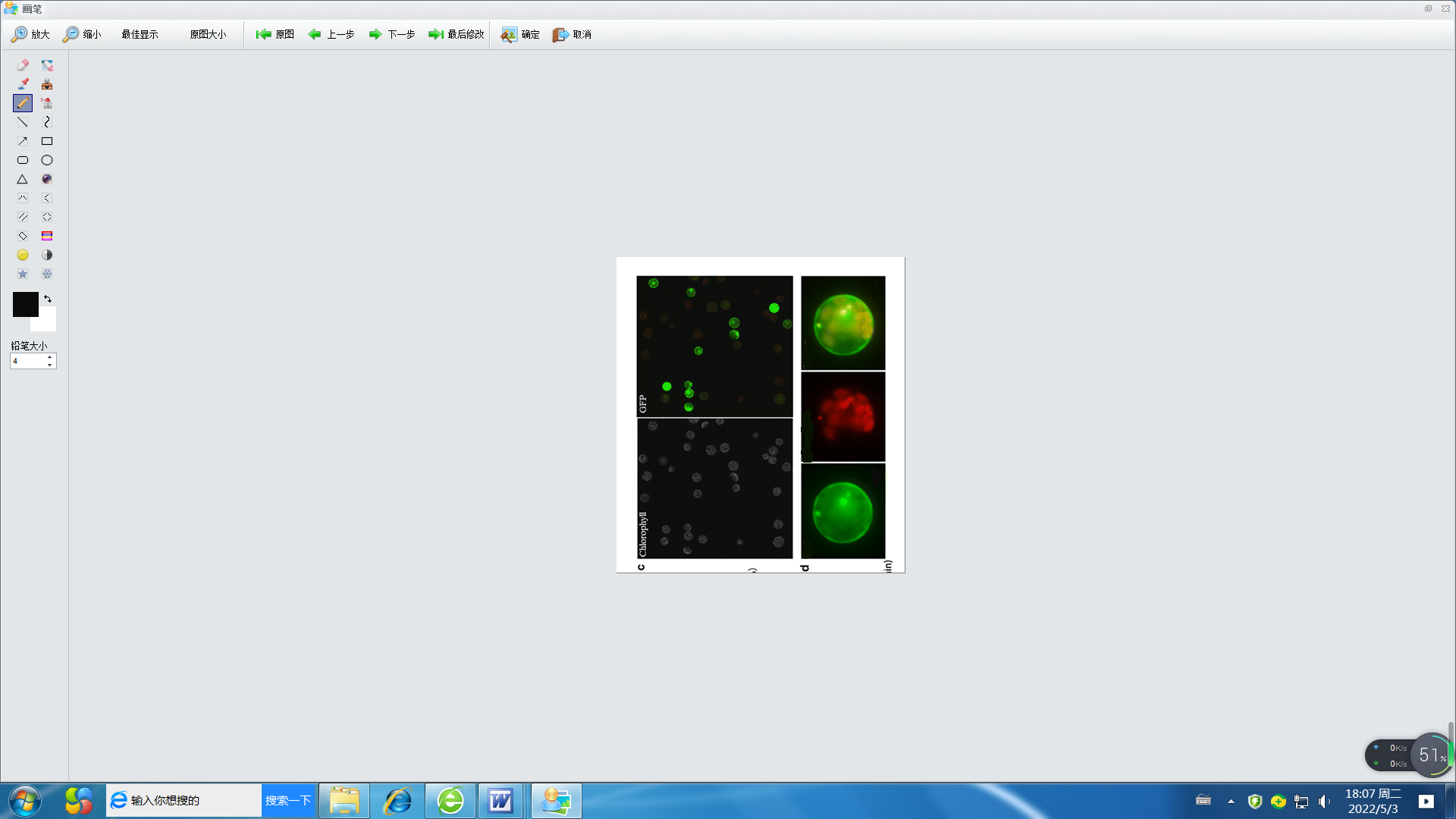

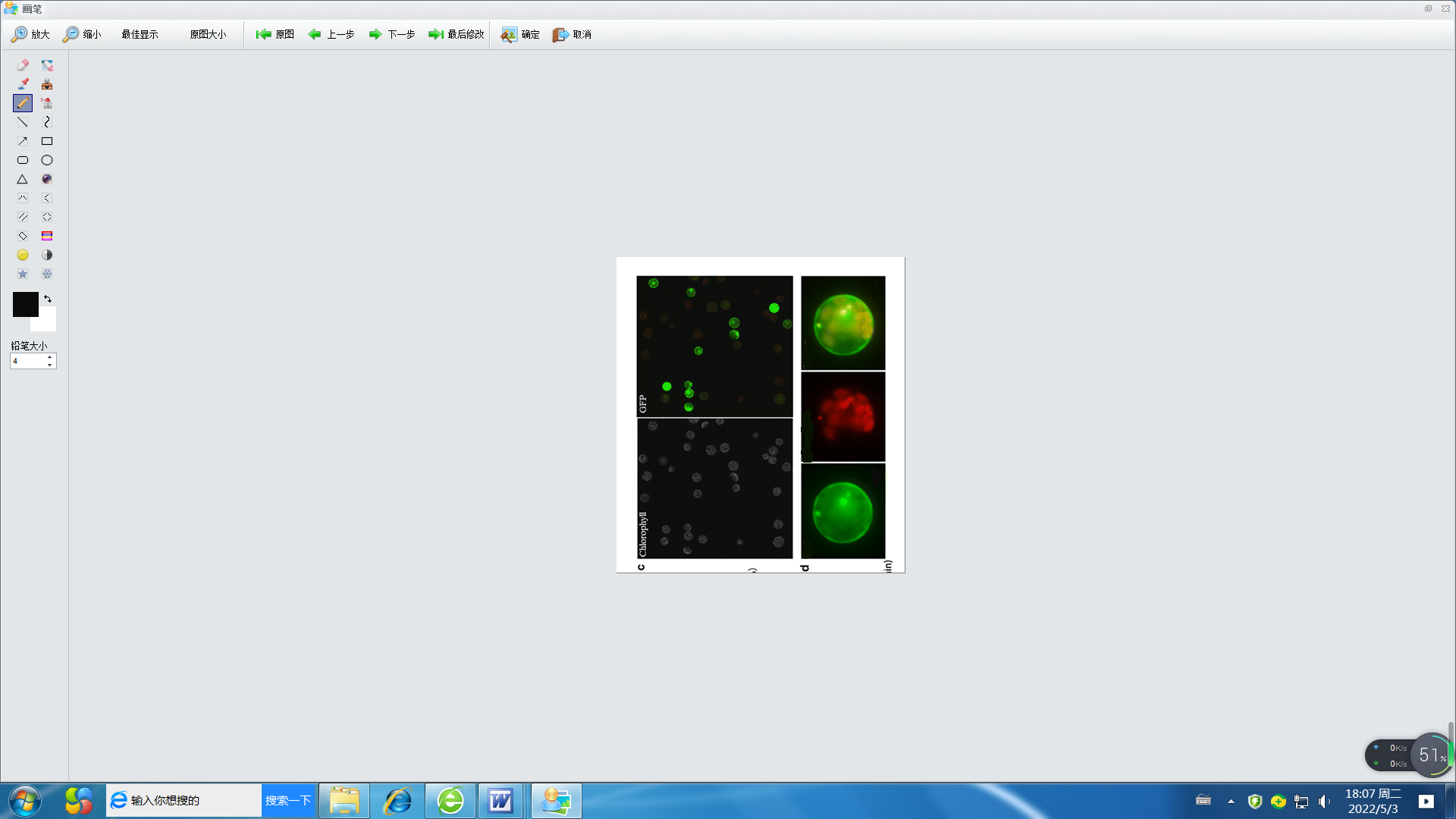


TaPYL4-GFP

Chlorophyll

Overlay

**Additional file 2** The signals initiated by TaPYL4-GFP in protoplast expression system of N. benthamiana observed under florescent microscope
