# Supplementary material for: TaPYL4, an ABA receptor gene of wheat, positively regulates plant drought adaptation through modulating the osmotic stress-associated processes
Source: BMC Plant Biol. 2022 Sep 1;22:423. doi: 10.1186/s12870-022-03799-z (PMC9434867; doi:10.1186/s12870-022-03799-z)
Supplement: Supplementary file 3 — Additional file 3. Expression levels and insertion copies of the target gene in TaPYL4 transgenic lines. [file 12870_2022_3799_MOESM3_ESM.docx]

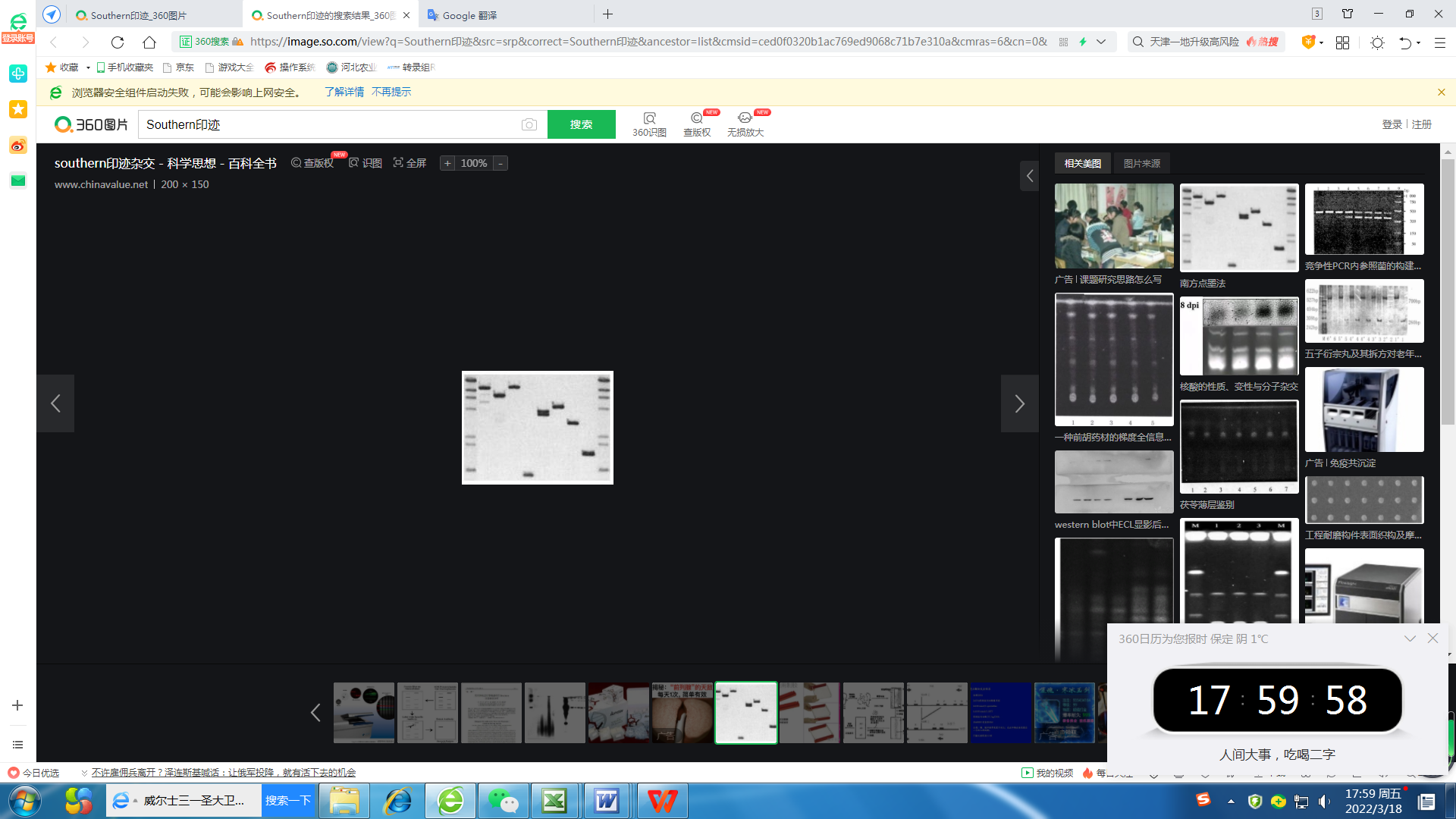


Sen 2 Sen 3 Anti 1 Anti 2 M

**B**

**A**

**Additional file 3** Expression levels insertion copies of the target gene in *TaPYL4* transgenic lines

**A**, Expression levels of target gene in transgenic lines; **B**, Insertion copies of target gene in transgenic lines subjected to gene functional analysis. WT, wild type; Sen 1 to Sen 6, transgenic lines with *TaPYL4* overexpression; Anti 1 to Anti 5, transgenic lines with *TaPYL4* knockdown expression. In **A**, Data are shown by average from triplicate results plus standard error with symbol * to represent statistically significant compared with WT (P<0.05). The expression values were normalized by *Tatubulin*, a constitutive gene in *T. aestivum* species.
