# Supplementary material for: TaPYL4, an ABA receptor gene of wheat, positively regulates plant drought adaptation through modulating the osmotic stress-associated processes
Source: BMC Plant Biol. 2022 Sep 1;22:423. doi: 10.1186/s12870-022-03799-z (PMC9434867; doi:10.1186/s12870-022-03799-z)
Supplement: Supplementary file 4 — Additional file 4. Stomata characterization on TaPYL4 transgenic lines of Sen 3 and Anti 2 upon drought stress. [file 12870_2022_3799_MOESM4_ESM.docx]

2 h

1 h

0.5 h

0 h

Anti 2

Sen 3

WT


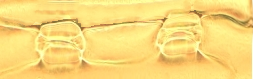

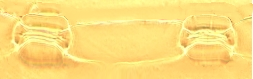

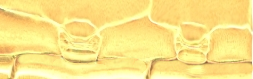

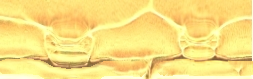

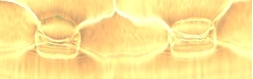

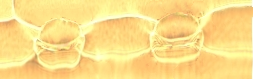

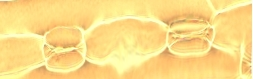

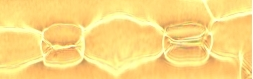

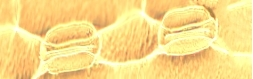

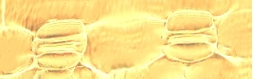

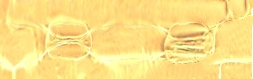

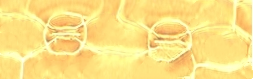


20μm

20μm

20μm

20μm

20μm

20μm

20μm

20μm

20μm

20μm

20μm

20μm

**A**

**B**

**Additional file 4** Stomata characterization on *TaPYL4* transgenic lines of Sen 3 and Anti 2 upon drought stress

**A**, stomata behaviors; **B**, stomata closing rates. Sen 3, a transgenic line with *TaPYL4* overexpression. Anti 2, a transgenic lines with *TaPYL4* knockdown expression. WT, wild type. In **B**, data shown are those relative to 0 h at time points after drought stress and symbol * represents statistical significance shown in transgenic lines compared with WT (P<0.05).
