# Supplementary material for: TaPYL4, an ABA receptor gene of wheat, positively regulates plant drought adaptation through modulating the osmotic stress-associated processes
Source: BMC Plant Biol. 2022 Sep 1;22:423. doi: 10.1186/s12870-022-03799-z (PMC9434867; doi:10.1186/s12870-022-03799-z)
Supplement: Supplementary file 5 — Additional file 5. Expression levels of the target gene detected in transgenic lines with TaP5CS1 knockdown expression. [file 12870_2022_3799_MOESM5_ESM.docx]

**Additional file 5** Expression levels of the target gene detected in transgenic lines with *TaP5CS1* knockdown expression

WT, wild type; AntiP5CS1-1 to AntiP5CS1-5, transgenic lines with *TaP5CS1* knockdown expression. Data are shown by average from triplicate results plus standard error with symbol * to represent statistically significant compared with WT (P<0.05). Expression values of target gene were normalized by *Tatubulin*, a constitutive gene in *T. aestivum* species.
