# Supplementary material for: TaPYL4, an ABA receptor gene of wheat, positively regulates plant drought adaptation through modulating the osmotic stress-associated processes
Source: BMC Plant Biol. 2022 Sep 1;22:423. doi: 10.1186/s12870-022-03799-z (PMC9434867; doi:10.1186/s12870-022-03799-z)
Supplement: Supplementary file 6 — Additional file 6. Expression levels of the target gene detected in transgenic lines with TaPIN9 knockdown expression. [file 12870_2022_3799_MOESM6_ESM.docx]

**Additional file 6** Expression levels of the target gene detected in transgenic lines with *TaPIN9* knockdown expression

WT, wild type; AntiPIN9-1 to AntiPIN9-5, transgenic lines with *TaPIN9* knockdown expression. Data are shown by average from triplicate results plus standard error with symbol * to represent statistically significant compared with WT (P<0.05). Expression values of target gene were normalized by *Tatubulin*, a constitutive gene.
