# Supplementary material for: TaPYL4, an ABA receptor gene of wheat, positively regulates plant drought adaptation through modulating the osmotic stress-associated processes
Source: BMC Plant Biol. 2022 Sep 1;22:423. doi: 10.1186/s12870-022-03799-z (PMC9434867; doi:10.1186/s12870-022-03799-z)
Supplement: Supplementary file 9 — Additional file 9. qRT-PCR results in roots for the differentially expressed genes with upregulated expression pattern identified based on RNA-seq analysis. [file 12870_2022_3799_MOESM9_ESM.docx]

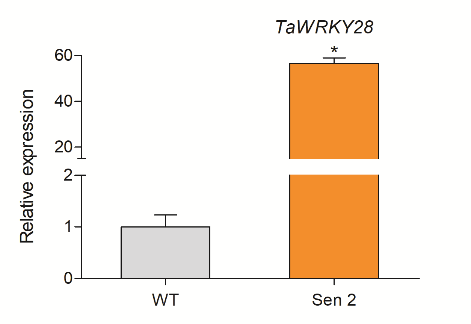

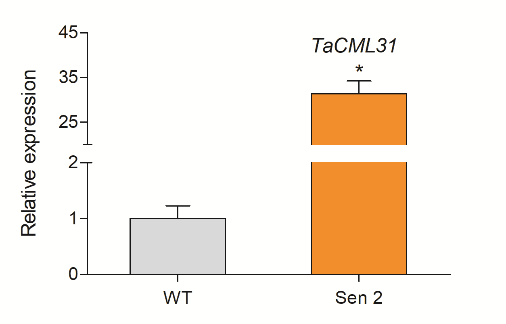

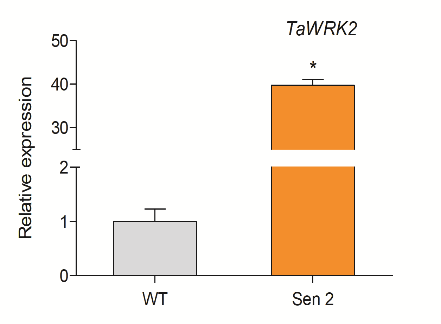

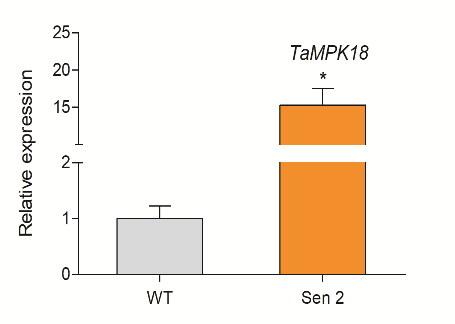

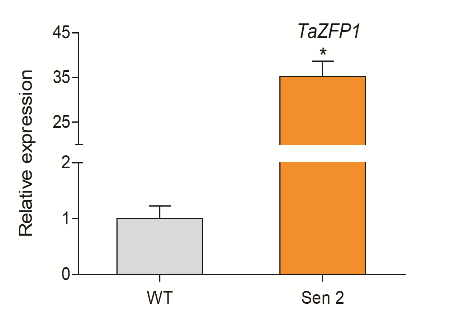

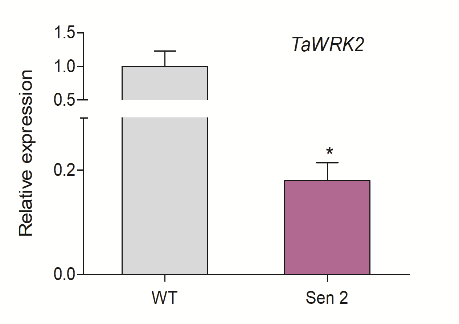

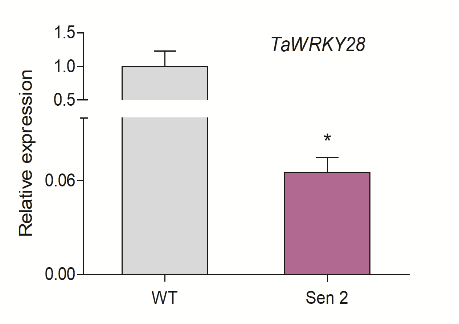

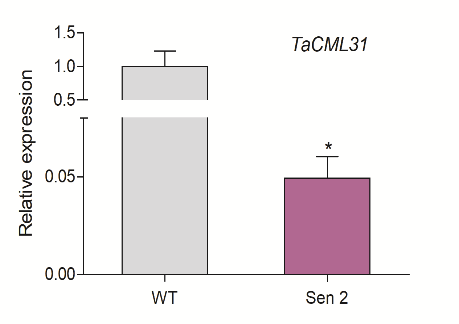

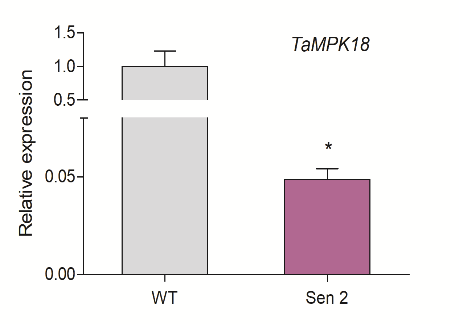

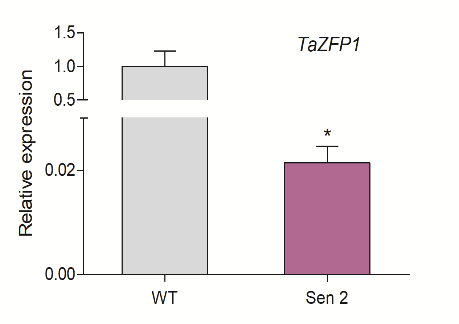


**A**

**B**

**Additional file 9** qRT-PCR results in roots for the differentially expressed genes with upregulated expression pattern identified based on RNA-seq analysis

**A**, expression levels in Sen 2; **B**, expression levels in Anti 1. In **A-B**, *TaWRK2*, wall-associated receptor kinase 2 (TraesCS3B02G007300), *TaWRKY28*, transcription factor WRKY28 (TraesCS7B02G418400), *TaCML31*, calcium-binding protein CML31 (TraesCS3B02G553900), *TaMPK18*, mitogen-activated protein kinase kinase kinase 18 (TraesCS3B02G288100), *TaZFP1*, zinc finger protein 1 (TraesCS5A02G401200). Sen 2, transgenic line overexpressing *TaPYL4*. Anti 1, transgenic line with *TaPYL4* knockdown expression. The constitutive gene *Tatubulin* was used as internal standard to normalize target gene transcripts. Average values are derived from triplicate results. Error bars represent standard errors and symbol * indicates significant differences between the transgenic lines and WT calculated by one-way ANOVA with significance level of 0.05.
