# Supplementary material for: TaPYL4, an ABA receptor gene of wheat, positively regulates plant drought adaptation through modulating the osmotic stress-associated processes
Source: BMC Plant Biol. 2022 Sep 1;22:423. doi: 10.1186/s12870-022-03799-z (PMC9434867; doi:10.1186/s12870-022-03799-z)
Supplement: Supplementary file 10 — Additional file 10. qRT-PCR results in roots for the differentially expressed genes with downregulated expression pattern identified based on RNA-seq analysis. [file 12870_2022_3799_MOESM10_ESM.docx]

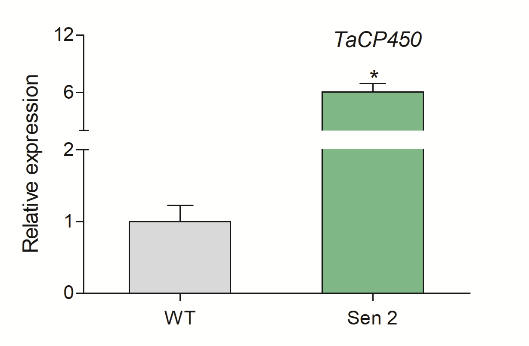

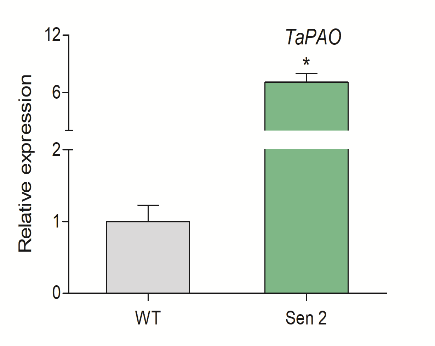

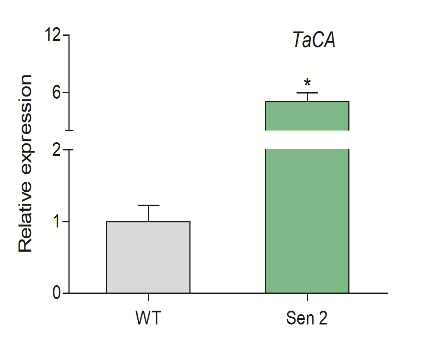

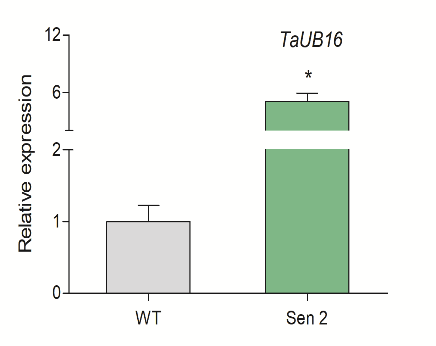

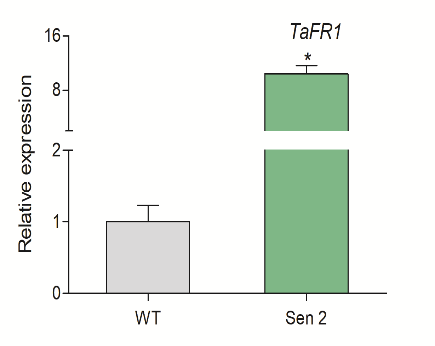

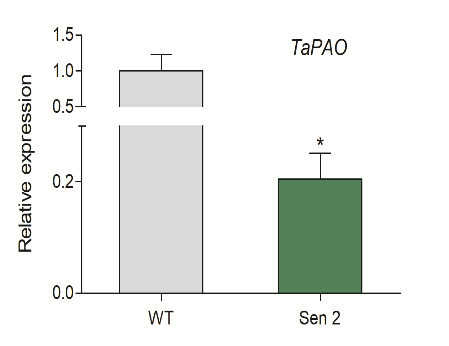

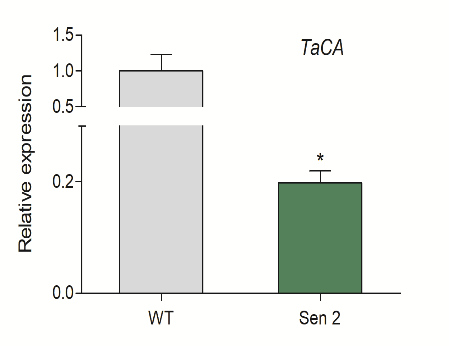

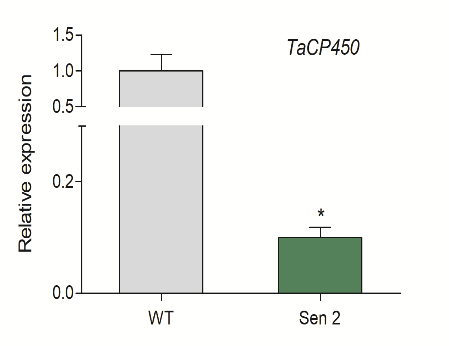

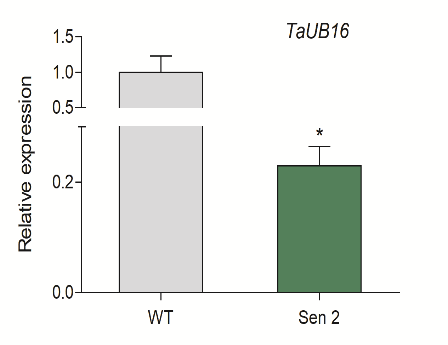

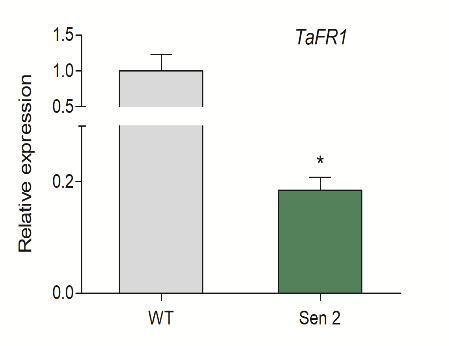


**A**

**B**

**Additional file 10** qRT-PCR results in roots for the differentially expressed genes with downregulated expression pattern identified based on RNA-seq analysis

**A**, expression levels in Sen 2; **B**, expression levels in Anti 1. In **A-B**, *TaPAO*, primary amine oxidase-like (TraesCS4A02G020900), *TaCA*, carbonic anhydrase (TraesCS3A02G230000), *TaCP450*, cytochrome P450 (TraesCS7D02G271100), *TaUBI6*, putative E3 ubiquitin-protein ligase SINA-like 6 (TraesCS3B02G288100), *TaFR1*, fatty acyl-CoA reductase 1-like (TraesCS3B02G016500). Sen 2, transgenic line overexpressing *TaPYL4*. Anti 1, transgenic line with *TaPYL4* knockdown expression. The constitutive gene *Tatubulin* was used as internal standard to normalize target gene transcripts. Average values are derived from triplicate results. Error bars represent standard errors and symbol * indicates significant differences between the transgenic lines and WT calculated by one-way ANOVA with significance level of 0.05.
