# Supplementary material for: TaPYL4, an ABA receptor gene of wheat, positively regulates plant drought adaptation through modulating the osmotic stress-associated processes
Source: BMC Plant Biol. 2022 Sep 1;22:423. doi: 10.1186/s12870-022-03799-z (PMC9434867; doi:10.1186/s12870-022-03799-z)
Supplement: Supplementary file 11 — Additional file 11. The cis-acting regulatory element ABRE identified in the TaPYL4 promoter region. [file 12870_2022_3799_MOESM11_ESM.docx]

**Additional file 11** The *cis*-acting regulatory element ABRE identified in the *TaPYL4* promoter region

| **Motif name** | **Organism** | **Position** | **Strand** | **Matrix score** | **Motif**  **sequence** | **Biological function** |
| --- | --- | --- | --- | --- | --- | --- |
| [ABRE](http://bioinformatics.psb.ugent.be/webtools/plantcare/cgi-bin/show_site_info.htpl?QWhere=ID_of_Site%20like%20'AACCCGG'&StartAt=0&NbRecs=10) | *Arabidopsis thaliana* | 1229 | + | 7 | AACCCGG | *cis*-acting element involved in the abscisic acid responsiveness |
| [ABRE](http://bioinformatics.psb.ugent.be/webtools/plantcare/cgi-bin/show_site_info.htpl?QWhere=ID_of_Site%20like%20'ACGTG'&StartAt=0&NbRecs=10) | *Arabidopsis thaliana* | 1679 | + | 5 | ACGTG | *cis*-acting element involved in the abscisic acid responsiveness |
| [ABRE](http://bioinformatics.psb.ugent.be/webtools/plantcare/cgi-bin/show_site_info.htpl?QWhere=ID_of_Site%20like%20'CGTACGTGCA'&StartAt=0&NbRecs=10) | *Hordeum vulgare* | 1676 | + | 9 | CGTACGTGCA | *cis*-acting element involved in the abscisic acid responsiveness |
| [ABRE](http://bioinformatics.psb.ugent.be/webtools/plantcare/cgi-bin/show_site_info.htpl?QWhere=ID_of_Site%20like%20'ACGTG'&StartAt=0&NbRecs=10) | *Arabidopsis thaliana* | 1702 | - | 5 | ACGTG | *cis*-acting element involved in the abscisic acid responsiveness |
