# Supplementary material for: TaPYL4, an ABA receptor gene of wheat, positively regulates plant drought adaptation through modulating the osmotic stress-associated processes
Source: BMC Plant Biol. 2022 Sep 1;22:423. doi: 10.1186/s12870-022-03799-z (PMC9434867; doi:10.1186/s12870-022-03799-z)
Supplement: Supplementary file 12 — Additional file 12. The yields of transgenic lines with overexpression of knockdown expression of TaPYL4 under normal irrigation and water-saving treatment in field experiment. [file 12870_2022_3799_MOESM12_ESM.docx]

**Additional file 12** The yields of transgenic lines with overexpression of knockdown expression of *TaPYL4* under normal irrigation and water-saving treatment in field experiment

Sen 2 and Sen 3, two lines with *TaPYL4* overexpression. Anti 1 and Anti 2, two lines with *TaPYL4* knockdown expression. WT, wild type. Normal irrigation, plants were irrigated at spring stages of jointing and mid-filling. Water-saving treatment, plants were irrigated at spring stage of jointing. The field experiment was conducted at Agricultural Experimental Station of Hebei Agricultural University, Badoing during 2021-2022 season. Values shown are averages derived from triplicate results and error bars indicate standard errors with symbol * to represent statistically significant among the transgenic lines and WT (P<0.05).
