# Supplementary material for: TaPYL4, an ABA receptor gene of wheat, positively regulates plant drought adaptation through modulating the osmotic stress-associated processes
Source: BMC Plant Biol. 2022 Sep 1;22:423. doi: 10.1186/s12870-022-03799-z (PMC9434867; doi:10.1186/s12870-022-03799-z)
Supplement: Supplementary file 13 — Additional file 13. Yeast two-hybrid assay results among TaPYL4, TaP5CS1 and TaPIN9 proteins. [file 12870_2022_3799_MOESM13_ESM.docx]

TaPYL4-BD+TaP5CS1-AD

TaPYL4-BD+TaPIN9-AD

TaP5CS1-BD+TaPIN9-AD

AD+ BD


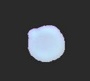

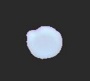

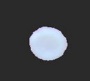

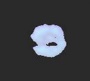

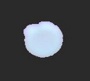

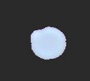

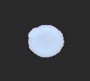

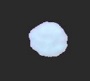

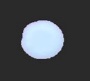

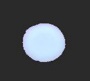

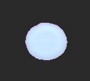

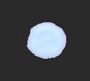


**1 10^-1^ 10^-2^**

**SD/-Trp/-Leu**

**1 10^-1^ 10^-2^**


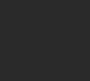

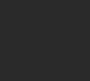

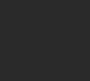

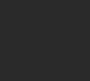

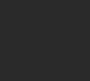

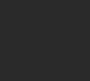

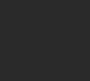

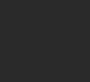

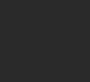

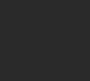

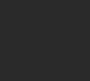

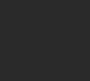


**SD/-Trp/-Leu/His**


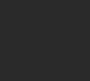

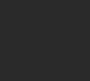

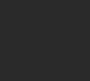

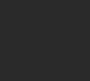

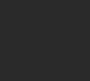

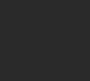

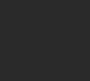

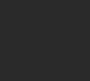

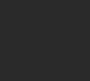

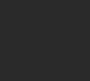

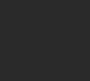

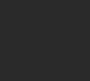


**1 10^-1^ 10^-2^**

**SD/-Trp/-Leu/His/Ade**


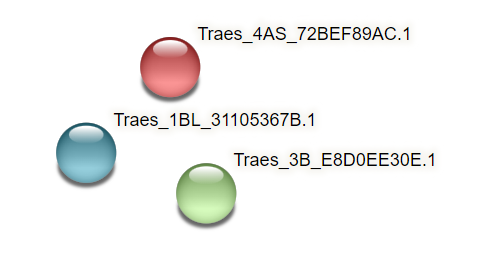


(TaPYL4)

(TaP5CS1)

(TaPIN9)

**A**

**B**

**Additional file 13** Yeast two-hybrid assay results among TaPYL4, TaP5CS1 and TaPIN9 proteins

**A**, protein interaction between TaPYL4 and TaP5CS1, protein interaction between TaPYL4 and TaPIN9, protein interaction between TaP5CS1 and TaPIN9; **B**, Protein-protein interaction prediction results among TaPYL4, TaP5CS1 and TaPIN9based on prediction tool STRING for protein-protein interaction (cn.string-db.org).
