# Supplementary material for: TaPYL4, an ABA receptor gene of wheat, positively regulates plant drought adaptation through modulating the osmotic stress-associated processes
Source: BMC Plant Biol. 2022 Sep 1;22:423. doi: 10.1186/s12870-022-03799-z (PMC9434867; doi:10.1186/s12870-022-03799-z)
Supplement: Supplementary file 14 — Additional file 14 Alignment results among TaPYL4 and its homeologs as well as expression of TaPYL4 homeologs upon drought stress. [file 12870_2022_3799_MOESM14_ESM.docx]

*TaPYL4*

*TaPYL4-4D*

*TaPYL4-4B*

*TaPYL4*

*TaPYL4-4D*

*TaPYL4-4B*

*TaPYL4*

*TaPYL4-4D*

*TaPYL4-4B*

*TaPYL4*

*TaPYL4-4D*

*TaPYL4-4B*

*TaPYL4*

*TaPYL4-4D*

*TaPYL4-4B*

**A**

**B**

**C**


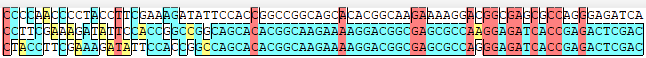

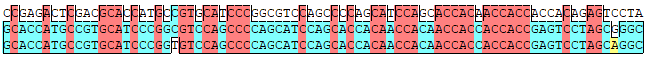

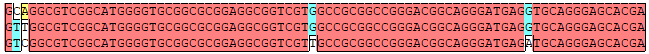

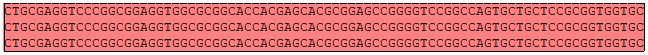

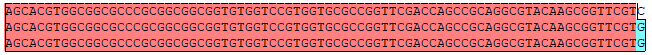

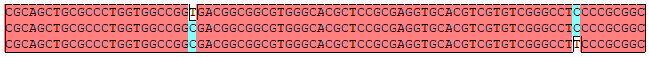

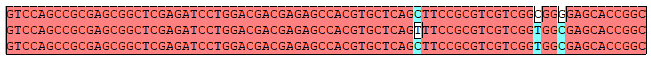

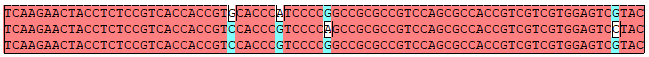

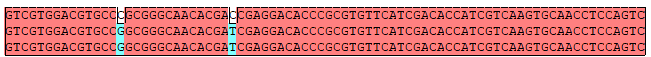

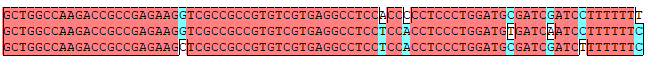

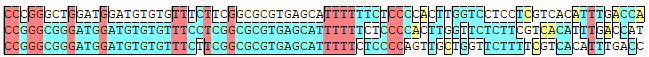

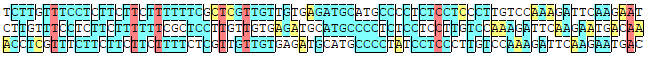

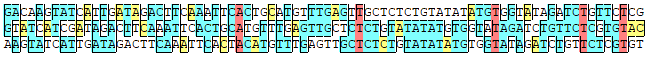

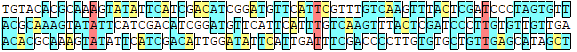


*TaPYL4*

*TaPYL4-4D*

*TaPYL4-4B*

initiation codon

termination codon


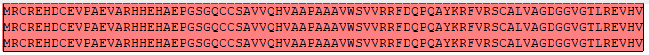

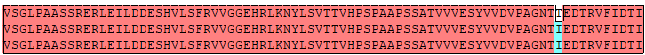

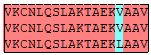


TaPYL4

TaPYL4-4D

TaPYL4-4B

*TaPYL4*

*TaPYL4-4D*

*TaPYL4-4B*

*TaPYL4*

*TaPYL4-4D*

*TaPYL4-4B*

*TaPYL4*

*TaPYL4-4D*

*TaPYL4-4B*

*TaPYL4*

*TaPYL4-4D*

*TaPYL4-4B*

*TaPYL4*

*TaPYL4-4D*

*TaPYL4-4B*

*TaPYL4*

*TaPYL4-4D*

*TaPYL4-4B*

*TaPYL4*

*TaPYL4-4D*

*TaPYL4-4B*

*TaPYL4*

*TaPYL4-4D*

*TaPYL4-4B*

TaPYL4

TaPYL4-4D

TaPYL4-4B

TaPYL4

TaPYL4-4D

TaPYL4-4B

**Additional file 14** Alignment results among *TaPYL4* and its homeologs as well as expression of *TaPYL4* homeologs upon drought stress

**A**, alignment results at nucleic acid level; **B**, alignment results at amino acid level; **C**, expression levels of *TaPYL4* and its homeologs in roots under normal growth and drought stress conditions. In **A** and **B**, the sequence alignment analyses were conducted based on MEGA8. In **C**, the roots of wheat seedlings (cv. Shimai 22) at the third-leaf stage grown in standard MS solution (control) and modified MS solution provided by PEG-6000 (10%, w/v) were collected to subjected to qRT-PCR analysis using specific primers for *TaPYL4* homeologs. *Tatubulin* was used as an internal standard. Average values are derived from triplicate results. Error bars represent standard errors and symbol * indicates significant differences between the transgenic lines and WT calculated by one-way ANOVA with significance level of 0.05..
