# Supplementary material for: TaPYL4, an ABA receptor gene of wheat, positively regulates plant drought adaptation through modulating the osmotic stress-associated processes
Source: BMC Plant Biol. 2022 Sep 1;22:423. doi: 10.1186/s12870-022-03799-z (PMC9434867; doi:10.1186/s12870-022-03799-z)
Supplement: Supplementary file 15 — Additional file 15. PCR primers used in this study. [file 12870_2022_3799_MOESM15_ESM.docx]

**Additional file 15**  PCR primers used in this study

| **Purpose** | **Accession number** | **Forward primer (5´-)** | **Reverse primer (5´-)** |
| --- | --- | --- | --- |
| *TaPYL4* expression | XM_044507276 | TCGTCGTG GAGTCGTACG | TCGATCGCATCCAGGGAGG |
| *Tatubulin* expression | U76558 | CATGCTATCCCTCGTCTCGACCT | CGCACTTCATGATGGAGTTGTAT |
| TaPYL4 location cassette | XM_044507276 | AAAACTAGTATGCCGTGCATCCCGGCG | AAAGGTACCACACGACACGGCGGCGAC |
| *TaPYL4* overexpression cassette | XM_044507276 | AAACCATGGCGTGCATCCCGGCGTC | AAAGGTAACCAGCCCGGGAAAAAAAG |
| *TaPYL4* knockdown expression cassette | XM_044507276 | AAACCATGGTGTCGATGAACACGC | AAAGGTAACCACCACCACAGAGTC |
| *TaP5CS1* expression | AB193551 | GCACGTGGACCTGTGGGTGTTG | GTTTTCGCGGAATCCTTACCACG |
| *TaP5CS2* expression | KM523670 | GGCCGTATACATGCACGTGGACCT | AGGTCCACGTGCATGTATACGG |
| *TaP5CS3* expression | KT868850 | CTCTTACGAGGGAAAGGGCAA | TCATTGCAAAGGAAGGCTC |
| *TaP5CS4* expression | KT218497 | CAAGTTGATAGGTATTTCTGAA | AATAAGGTATCTGTTGCCTCAA |
| *TaP5CS5* expression | AY888045 | TGGTCACTACAGATGATAAAGT | TACTTATGCCAACCTCAGCACC |
| *TaP5CS1* knockdown expression cassette | AB193551 | TTTCCATGGTGAGACTCTCATACTT | TTTTTGTACCGCAAGCTCGTCAAT |
| *TaPIN1* expression | AY496058 | CCCAGGGCAT TGTCCCCTT | AGGCCGAGCAGGATGTAGTA |
| *TaPIN2* expression | XM_044578249 | CCACCAGTTGATTCTTCTGTC | TGGGAACCGCATGTATTAGT |
| *TaPIN3* expression | XM_044493290 | GGGGAGCCAGCCTATAGCA | GGCATCGAGGACACGCTACA |
| *TaPIN5* expression | XM_044581091 | GCCCGTACTCATCGGGTATT | AGTATTGTCCCATTGAGGAGC |
| *TaPIN8* expression | XM_044497417 | GGCCAAACAATAGCA GGGCA | CTTTATGTCGGGTCACCACA |
| *TaPIN9* expression | XM_044579445 | GACGTTGTTCGCATAGGATTA | TAGGTGACCAATACACAGGCC |
| *TaPIN9* knockdown expression cassette | XM_044579445 | AAACCATGGAATGAGATAAGGATCC | AAAGGTGACCCCTACGCCATGAAC |
| TaPYL4 bait in two-hyb | XM_044507276 | TTTGAATTCATGCCGTGCATCCCGGCG | TTTCTGCAGATCACGACACGGCGGCGA |
| TaPP2C1 prey in two-hyb | TraesCS1A02G358600 | TTTGAATTCATGGCGGCGGCGGCGGCG | TTTCTGCAGGTCAGCTTTGCTCATCCG |
| TaPP2C2 prey in two-hyb | TraesCS1A02G441200 | TTTGAATTCATGGACGCCCTAGGCGCC | TTTCTGCAGGCTATCTGTTGTTGTTGT |
| TaPP2C3 prey in two-hyb | TraesCS1B02G375100 | TTTGAATTCATGGCGGCGGCGGCGGCG | TTTCTGCAGGTTAGGAATTATTCTTGG |
| TaPP2C4 prey in two-hyb | TraesCS1D02G363500 | TTTGAATTCATGGCGGCGGCGGCGGCC | TTTCTGCAGGTTAGGAATTATTCTTGG |
| TaPP2C5 prey in two-hyb | TraesCS1A02G441200 | TTTGAATTCATGGACGCCCTAGGCGCC | TTTCTGCAGGCTATCTGTTGTTATTGT |
| TaPP2C6 prey in two-hyb | TraesCS3B02G240000 | TTTGAATTCATGGAGGACGTGGCCGTG | TTTCTGCAGGTTAAGTTTTGCTCTTGA |
| TaPP2C2 bait in two-hyb | TraesCS1A02G441200 | TTTGAATTCATGGACGCCCTAGGCGCC | TTTCTGCAGACTATCTGTTGTTGTTGT |
| TaSnRK2.1 prey in two-hyb | TraesCS2A02G493800 | TTTGAATTCCATGGATCGGTACGAGGTG | TTTGGATCCTCACAACGGGCACACGAAA |
| TaSnRK2.2 prey in two-hyb | TraesCS2A02G163800 | TTTGAATTCATGGAGCGGTACGAGGTG | TTTGGATCCTCACAACGCGCACACGAA |
| TaSnRK2.3 prey in two-hyb | TraesCS1A02G215900 | TTTGAATTCATGGAGGAGAGGTACGAG | TTTGGATCCTCAGTAGGTCTCCCCCTC |
| TaSnRK2.4 prey in two-hyb | TraesCS3A02G381100 | TTTGAATTCATGGAGAAGTACGAGGCG | TTTGGATCCTCATGATATGCGTAGCGA |
| TaSnRK2.5 prey in two-hyb | TraesCS2A02G566700 | TTTGAATTCATGGAGAAGTACGAGCCGG | TTTGGATCCTCAGATTTGGAGCTTGCT |
| TaSnRK2.6 prey in two-hyb | TraesCS2A02G303900 | TTTGAATTCATGGAGAGGTACGAGCTG | TTTGGATCCCTAGCTGATGTGGAACTC |
| TaSnRK2.7 prey in two-hyb | TraesCS1A02G270800 | TTTGAATTCATGGACAAGTACGAGGAGG | TTTGGATCCTTAGATGTGCAACACGCT |
| *TaWRK2* | TraesCS3B02G007300 | GGTGCTGCCAGACTGCCAT | TCTCCGGTGAAGGTGTTGTTG |
| *TaWRKY28* | TraesCS7B02G418400 | GCCAGCATAGGAAAATATAGT | GGTTCCCATGGCCTCAATTTG |
| *TaCML31* | TraesCS3B02G553900 | GCTACCTAGCGCCAGGATCCA | ACGGACAATCCATCGAGGCC |
| *TaMPK18* | TraesCS3B02G288100 | GCACAAATTTCCTTCTGTCTTGA | AGGCTATAAGCAGCAAGCAG |
| *TaZFP1* | TraesCS5A02G401200 | CCGAGTCCGAGGTGGGGAGC | AGCCGGGGCTTCTTGAAGGC |
| *TaPAO* | TraesCS4A02G020900 | CCACGAGACCGCCGGGCTT | TCTGTTGTGGGTTCCAGAGC |
| *TaCA* | TraesCS3A02G230000 | CCCCAGCGGTTAACTCCTA | TCCCGGCATCCAGTACTGG |
| *TaCP450* | TraesCS7D02G271100 | TGGCTGGGCCATGCGGGC | TGATAGTAGCTCATAACTACGC |
| *TaUBI6* | TraesCS3B02G28810 | GGACAGAGCCTTTTGGGCCAC | ACAACAAGCTACGTACATAGC |
| *TaFR1* | TraesCS3B02G016500 | CCCAGTGCGGTAACTATCCA | CATCTGGCAAACACTACACC |
